# Preclinical Evidence of Anti-Tumor Activity Induced by EZH2 Inhibition in Human Models of Synovial Sarcoma

**DOI:** 10.1371/journal.pone.0158888

**Published:** 2016-07-08

**Authors:** Satoshi Kawano, Alexandra R. Grassian, Masumi Tsuda, Sarah K. Knutson, Natalie M. Warholic, Galina Kuznetsov, Shanqin Xu, Yonghong Xiao, Roy M. Pollock, Jesse S. Smith, Kevin K. Kuntz, Scott Ribich, Yukinori Minoshima, Junji Matsui, Robert A. Copeland, Shinya Tanaka, Heike Keilhack

**Affiliations:** 1 Epizyme Inc., Cambridge, Massachusetts, United States of America; 2 Eisai Co., Ltd., Tsukuba, Ibaraki, Japan; 3 Eisai Inc., Andover, Massachusetts, United States of America; 4 Department of Cancer Pathology, Hokkaido University Graduate School of Medicine, Sapporo, Hokkaido, Japan; Ospedale Pediatrico Bambino Gesu', ITALY

## Abstract

The catalytic activities of covalent and ATP-dependent chromatin remodeling are central to regulating the conformational state of chromatin and the resultant transcriptional output. The enzymes that catalyze these activities are often contained within multiprotein complexes in nature. Two such multiprotein complexes, the polycomb repressive complex 2 (PRC2) methyltransferase and the SWItch/Sucrose Non-Fermentable (SWI/SNF) chromatin remodeler have been reported to act in opposition to each other during development and homeostasis. An imbalance in their activities induced by mutations/deletions in complex members (e.g. *SMARCB1*) has been suggested to be a pathogenic mechanism in certain human cancers. Here we show that preclinical models of synovial sarcoma—a cancer characterized by functional SMARCB1 loss via its displacement from the SWI/SNF complex through the pathognomonic SS18-SSX fusion protein—display sensitivity to pharmacologic inhibition of EZH2, the catalytic subunit of PRC2. Treatment with tazemetostat, a clinical-stage, selective and orally bioavailable small-molecule inhibitor of EZH2 enzymatic activity reverses a subset of synovial sarcoma gene expression and results in concentration-dependent cell growth inhibition and cell death specifically in SS18-SSX fusion-positive cells *in vitro*. Treatment of mice bearing either a cell line or two patient-derived xenograft models of synovial sarcoma leads to dose-dependent tumor growth inhibition with correlative inhibition of trimethylation levels of the EZH2-specific substrate, lysine 27 on histone H3. These data demonstrate a dependency of SS18-SSX-positive, SMARCB1-deficient synovial sarcomas on EZH2 enzymatic activity and suggests the potential utility of EZH2-targeted drugs in these genetically defined cancers.

## Introduction

Posttranslational modification of histone proteins and chromatin remodeling are crucial processes for the regulation of the fidelity of normal gene expression. There are many proteins involved in these processes, including protein methyltransferases (PMTs) and ATP-dependent chromatic remodelers [1, 2]. In cells these enzymes are often presented as components of compulsory multiprotein complexes (i.e., enzymatic activity requires complex formation). Two such complexes are the Polycomb Repressive Complex 2 (PRC2) and SWItch/Sucrose Non-Fermentable (SWI/SNF) Complex. Importantly, these two complexes normally compete with each other for binding to chromatin and have opposing functions: the PRC2 complex (of which EZH2 is the catalytic subunit) is responsible for the trimethylation of histone 3 at lysine 27 (H3K27Me3), leading to repression of gene expression, whereas the SWI/SNF complex is an ATP-dependent chromatin remodeler which allows for activation of gene transcription [3]. Interestingly, members of both of these complexes are frequently genetically altered in cancer and these mutations can create an imbalance in the antagonistic function between these two complexes [4, 5]. For instance, SMARCB1 (INI1/SNF5/BAF47) is a subunit of the SWI/SNF complex that is lost in nearly all rhabdoid tumors, creating an oncogenic dependency on the PRC2-EZH2 methyltransferase, and sensitizing these tumors to small molecule inhibitors of EZH2 both *in vitro* and *in vivo* [6, 7]. The loss of SMARCB1 results in unregulated PRC2-mediated gene repression in these tumors, thus preventing the de-repression of genes involved in differentiation and other anti-tumorigenic functions. As such, inhibitors of EZH2 are now being tested in the clinic, and objective responses have been observed in SMARCB1-negative rhabdoid tumors in the setting of a Phase I study [8].

Additional mutations in the SWI/SNF complex are observed in a variety of tumor types, including mutations in *SMARCA4*, *SMARCA2*, *ARID1A* and others [9]. In synovial sarcoma a recurrent chromosomal translocation fuses the *SS18* gene (a subunit of the SWI/SNF chromatin remodeling complex [10]) on chromosome 18 to one of three related genes on the X chromosome, *SSX1*, *SSX2* and rarely *SSX4*, resulting in the expression of a SS18-SSX fusion protein [11–16]. This translocation is believed to be the sole driving event in synovial sarcomas [17], as expression of the translocation product in mouse models induces synovial sarcoma tumors at 100% penetrance [18], and expression of the translocation in synovial sarcoma cell lines is required for their survival [19]. Additionally, the *SS18-SSX* translocation is frequently the sole chromosomal aberration observed in synovial sarcoma, and there are very few additional mutations present in this indication [17].

Functionally, it has been shown that the SS18-SSX fusion protein is still incorporated into the SWI/SNF complex, with concomitant eviction of the SMARCB1 subunit [9]. The SMARCB1 protein may be relatively unstable in the absence of binding to the SWI/SNF complex, as the levels of SMARCB1 expression are greatly reduced in the presence of the SS18-SSX fusion [9]. This generates a state of SMARCB1-deficiency without mutations within the *SMARCB1* gene itself. SMARCB1 expression can be rescued by knockdown of the fusion protein or by overexpressing wild-type *SS18* (which competes for incorporation into the SWI/SNF complex) further confirming the misregulation of SMARCB1 expression induced by this translocation [9].

Given the sensitivity of SMARCB1-deficient tumors to EZH2 inhibition, and the diminution of SMARCB1 levels observed in SS18-SSX translocation positive synovial sarcomas, we hypothesized that synovial sarcomas may be similarly dependent on PRC2/EZH2 activity. Here we show that tazemetostat (formerly known as EPZ-6438 or E7438), an early clinical-stage, selective and orally bioavailable small molecule inhibitor of EZH2 enzymatic activity induces anti-proliferative activity in preclinical models of synovial sarcoma, both as a single agent and in combination with chemotherapy. We observe that synovial sarcoma cell lines are sensitive to inhibition of EZH2, and that this sensitivity correlates with alteration of expression of certain genes previously implicated in synovial sarcoma. A dependence on EZH2 activity is similarly observed in a cell line xenograft model as well as in two patient-derived xenograft (PDX) models, with correlative inhibition of trimethylation levels of the EZH2-specific substrate, H3K27. Thus, these data demonstrate a sensitivity of SS18-SSX-positive synovial sarcomas to inhibition of EZH2 enzymatic activity and support the further investigations in the clinical setting.

## Materials and Methods

### Cell culture

Human synovial sarcoma line HS-SY-II was obtained from Riken BioResource Center. Human synovial sarcoma line Fuji was established as described previously [20]. Human soft tissue sarcoma cell line SW982 (HTB-93) was obtained from American Type Culture Collection. All cell lines were cultured in RPMI + 10% FBS. For examination of changes in H3K27Me3 and gene expression, each of the cell lines were plated to ensure cell densities were within linear log phase growth until sample collection. Cells were treated with either DMSO or tazemetostat as indicated. After the treatment for 96 hours (H3K27Me3 analysis) or at each time point (gene expression analysis), cells were washed with PBS. For analysis of H3K27Me3 alterations, cells were harvested and subjected to histone extraction. For analysis of gene expression alterations, cells were lysed using a Cells-to-Ct kit (Applied Biosystems) according to the manufacturer's protocol.

### Immunoblot

Protein concentrations were determined by BCA Protein assay (Pierce). A sample solution was prepared by mixing 2 × loading buffer (β-ME Sample Treatment for Tris SDS, Cosmo bio) and water with cell lysates or extracted histones, and incubated for 5 minutes at 95°C. Immunoblot analysis was performed as follows. The sample solutions were separated on 15–25% (for histones) or 4–20% (for other proteins) gradient agarose gel under reducing conditions and transferred to nitrocellulose membranes (GE Healthcare) and probed with the following antibodies: rabbit polyclonal anti-EZH2 antibody (07–689, Millipore), rabbit monoclonal anti-SMARCB1 antibody (CST 8745), mouse monoclonal anti-SS18 antibody (sc-365170, Santa Cruz), mouse monoclonal anti-β-actin antibody (A5441, Sigma-Aldrich), rabbit monoclonal anti-H3K27Me3 antibody (CST 9733), rabbit monoclonal anti-H3K27Me2 antibody (CST 9728), and rabbit polyclonal anti-total H3 antibody (ab1791, Abcam). Immunoblotting was performed on an iBind Western Device (Life Technologies) according to the manufacturer's instructions using horseradish peroxidase conjugated anti-rabbit IgG or anti-mouse IgG antibodies (Cell Signaling Technology). Blots were developed with Immobilon Western chemiluminescent HRP substrate (Millipore). Immunoreactive bands were visualized by chemiluminescence with Luminescent Image Analyzer LAS-3000 (Fuji Film) and the signals of protein bands were quantified using Multi Gauge version 3.0 software (Fuji Film).

### Proliferation assays

The cell lines were harvested with 0.25% trypsin solution, counted, diluted, and dispensed at 100 μL/well in collagen type 1-treated 96-well plates (IWAKI) for Fuji cells or tissue culture treated 96-well plates (FALCON) for HS-SY-II and SW982 cells. The cells were also dispensed at 2 mL/well in collagen type 1-treated 6-well plates (IWAKI) for Fuji cells or tissue culture treated 6-well plates (FALCON) for HS-SY-II and SW982 cells. Each of the cell lines were plated to ensure cell densities were within linear log phase growth until measuring cell viability or the cells were replated (Fuji, 500 cells in 96-well and 15,000 cells in 6-well; HS-SY-II, 800 cells in 96-well and 24,000 cells in 6-well; SW982, 250 cells in 96-well and 7500 cells in 6-well). The cells were incubated under 37°C, 5% CO_2_ condition (day 0). Several hours later, 100 μL or 2 mL of culture medium containing tazemetostat or 0.2% of DMSO as control was added to each well of 96-well or 6-well plates to yield final concentrations of 0 (control), 0.039, 0.16, 0.63, 2.5, and 10 μmol/L with final concentration of DMSO at 0.1% for measurement of cell viability on days 4 and 7. These 96-well plates were used for measuring cell viability on day 0, 4, and 7. On day 7, the cells in the 6-well plates were harvested with 0.25% trypsin solution and counted. The cells were replated in 96-well plates at the same density of cells in 96-well plates on day 0, and 100 μL of tazemetostat or DMSO control was added in the plates as conducted on day 0 for measurement of cell viability on days 11 and 14. Compound/media was changed with new one on days 4 and 11 at concentrations of 0 (control), 0.039, 0.16, 0.63, 2.5, and 10 μmol/L. On days 0, 4, 7, 11, and 14, cell viability was determined by measuring ATP contents by CellTiter-Glo^®^ Luminescent Cell Viability Assay (Promega) with EnVision 2102 Multilabel Reader (PerkinElmer). The ratios of the measured values on days 4 and 7 to that of day 0, and days 11 and 14 to that of replated day 7 were used. Overall plot of proliferation of day 11 or day 14 was calculated (ratio of day 7) × (ratio of day 11 or ratio of day 14). Three independent experiments were performed in triplicate. The mean 50% inhibitory concentration (IC_50_) value and 95% confidence interval (CI) were calculated based on the IC_50_ values generated from separate curves representing the growth activity versus tazemetostat concentration of 3 independent experiments. Statistical analyses were performed using the GraphPad Prism version 6.02 (GraphPad Software).

### Cell cycle and apoptosis assays

Fuji and HS-SY-II cells were incubated with tazemetostat at concentration of each cell line’s IC_50_, 0.15 μmol/L and 0.52 μmol/L, respectively, for 4, 7, 11, and 14 days. Cell cycle analysis was performed after labeling DNA with propidium iodide. Apoptosis assay was performed using a FITC Annexin V Apoptosis Detection Kit I (BD Biosciences) and Caspase-Glo^®^ 3/7 Assay (Promega) according to the manufacturer's protocol. All FACS analysis was performed on an LSR Fortessa flow cytometer (BD Biosciences). Caspase 3/7 activity was determined with EnVision 2102 Multilabel Reader (PerkinElmer).

### Xenograft studies

All the procedures related to animal handling, care, and the treatment in this study were performed according to the guidelines approved by the Institutional Animal Care and Use Committee (IACUC) of Champions Oncology and Eisai Tsukuba following the guidance of the Association for Assessment and Accreditation of Laboratory Animal Care.

HS-SY-II xenograft studies were performed at Eisai Tsukuba (pharmacodynamics study) and HAMRI Tsukuba Research center (efficacy study). The protocols were approved by the IACUC of Eisai Tsukuba (Permit Number: 13-D-0307-001 for efficacy study and 13-D-0154-001 for pharmacodynamics study). HS-SY-II cells were harvested during mid-log phase growth, and resuspended in PBS with 50% Matrigel (BD Biosciences). Balb/C-nu mice (Charles River Laboratories Japan) received 1 × 10^7^ cells (0.1 mL cell suspension) subcutaneously in the right flank. Mice carrying tumors of approximately 170 mm^3^ for efficacy study (35 days after injection) or 220 mm^3^ for pharmacodynamics study (31 days after injection) were sorted into treatment groups with similar mean tumor volumes. Tazemetostat or vehicle (0.5% methyl cellulose + 0.1% Tween-80 in water) was administered at the indicated doses on twice a day schedules for either 7 or 28 days by oral gavage. As reference compound, doxorubicin HCl in saline was dosed at 10 mg/kg. Each dose was delivered in a volume of 0.2 mL/20 g mouse (10 mL/kg), and adjusted for the last recorded weight of individual animals. Mice were monitored for overall health status daily and their tumor volumes were monitored twice a week using a digital caliper throughout the experiment. On day 7 (pharmacodynamics study) or day 28 (efficacy study), mice were sampled in a prespecified fashion. Sampling included nonterminal bleeds (0.15 mL) from the retro-orbital venous plexus with anesthesia by isoflurane inhalation. Blood samples were processed for plasma, with Na-heparin as anticoagulant. The plasma samples were frozen at −80°C and stored before bioanalysis of compound levels. Tumors were harvested from specified mice. Tumor tissue from each animal was frozen at −80°C or stored in RNAlater (Ambion) and kept at −20°C until total RNA purification was performed.

Fuji xenograft studies were performed at Eisai Tsukuba. The protocols were approved by the IACUC of Eisai Tsukuba (Permit Number: 13-D-0307-002 for efficacy study and 14-D-0047-001 for pharmacodynamics study). Fuji cells were harvested during mid-log phase growth, and resuspended in HBSS with 50% Matrigel. Balb/C-nu mice received 1 × 10^7^ cells (0.1 mL cell suspension) subcutaneously in the right flank. Mice carrying tumors of approximately 200 mm^3^ for efficacy study (25 days after injection) or 260 mm^3^ for pharmacodynamics study (26 days after injection) were sorted into treatment groups with similar mean tumor volumes. Tazemetostat, EPZ011989 or vehicle (0.5% methylcellulose + 0.1% Tween-80 in water) was administered at the indicated doses on twice a day schedules for either 7, 29 or 35 days by oral gavage. Due to reaching tumor volume endpoint on 2000 mm^3^ some of the mice in the vehicle group needed to be euthanized on day 14. As reference compound, doxorubicin HCl in saline was dosed at 10 mg/kg. Each dose was delivered in a volume of 0.2 mL/20 g mouse (10 mL/kg), and adjusted for the last recorded weight of individual animals. Mice were monitored for overall health status daily during the dosing period or twice a week after the dosing period, and their tumor volumes were monitored twice a week using a digital caliper throughout the experiment. On day 7 of the pharmacodynamics study, mice were sampled in a prespecified fashion. Sampling included nonterminal bleeds (0.15 mL) from the retro-orbital venous plexus with anesthesia by isoflurane inhalation. Blood samples were processed for plasma, with Na-heparin as anticoagulant. The plasma samples were frozen at −80°C and stored before bioanalysis of compound levels. Tumors were harvested from specified mice. Tumor tissue from each animal was frozen at −80°C or stored in RNAlater (Ambion) and kept at −20°C until total RNA purification was performed.

PDX studies (CTG-0771, CTG-0331, and CTG-1169) were performed at Champions Oncology. The protocols were approved by the IACUC of Champions Oncology (Permit Number: CHA-003-2013). Tumor fragments (5 mm x 5 mm x 5 mm) were harvested from donor animals, each implanted from a specific passage lot (CTG-0331 and CTG-0771 at P4). NCr-nu mice (Taconic) received the fragment subcutaneously in the flank region. Mice carrying tumors of approximately 100–300 mm^3^ were sorted into treatment groups with similar mean tumor volumes. Tazemetostat or vehicle (0.5% sodium carboxymethylcellulose +0.1%Tween-80 in water) was administered at the indicated doses on twice a day schedules for 35 days by oral gavage. As reference compound, doxorubicin HCl in saline was dosed at 3 mg/kg. Each dose was delivered in a volume of 0.2 mL/20 g mouse (10 mL/kg), and adjusted for the last recorded weight of individual animals. Mice were monitored for overall health status daily and their tumor volumes were monitored twice a week using a digital caliper throughout the experiment. For the CTG-0771 and CTG-0331 models, 3 to 5 mice per group with the largest tumors were euthanized by carbon dioxide inhalation on day 35 for blood and tissue sampling, and the 7 remaining mice were followed for an additional 25 days as animal survival study. Analgesics and anesthetics were not administered. Mice were humanely euthanized by carbon dioxide inhalation if their body weights dropped more than 20% of the original weight for two consecutive measurements, more than 30% of the original weight for one measurement, their xenograft tumor grew larger than 2000 mm^3^, or they showed appear moribund at any time, while a tumor volume endpoint in Kaplan-Meier survival curve was 1200 mm^3^, with the exception of the slower growing CTG-1169 model for which 600 mm^3^ was used as an endpoint for Kaplan-Meier survival curve Diet gel was provided *ad libitum* if their body weights dropped more than 10%. The summary of deaths for the 3 models is as follows: For CTG-0771 there was one animal found dead in 400–500 mg/kg group. For CTG-0331 there was no dead animal. For CTG-1169 there were four animals that were found dead in multiple groups, two animals in 125 mg/kg group, one animal in 500 mg/kg group, and one animal in doxorubicin group. For the above cases, necropsies were performed and the causes of death were not clarified.

In all animal studies, food and water were available *ad libitum*. Detailed information about the human tumors that were the source for the 3 PDX models are shown in Table 1.

**Table 1:** Detailed information about human tumors used to generate PDX models.

Model	Tumor	Patient Gender	Patient Age	Type	Passage
CTG-0331	Synovial Sarcoma (SSX2 Translocation)	Female	16	Metastatic	4
CTG-0771	Synovial Sarcoma (SSX2 Translocation)	Male	57	Metastatic	4
CTG-1169	Synovial Sarcoma (SSX1 Translocation)	Male	51	Metastatic	5

### RNA-seq Analysis

RNA extraction was performed as previously described [21]. RNA samples were converted into cDNA libraries using the Illumina TruSeq Stranded mRNA sample preparation kit (Illumina RS-122-2103). Briefly, Total RNA samples are concentration normalized, and polyadenylated RNA is purified using oligo-dT attached to magnetic beads. Purified mRNA is fragmented using heat in the presence of divalent cations. Fragmented RNA is converted into double-stranded cDNA, with dUTP utilized in place of dTTP in the second strand master mix. A single 'A' base is added to the cDNA and forked adaptors that include index, or barcode, sequences are attached via ligation. The resulting molecules are amplified via polymerase chain reaction (PCR). During PCR the polymerase stalls when a dUTP base is encountered in the template. Since only the second strand includes the dUTP base, this renders the first strand the only viable template, thereby preserving the strand information. Final libraries are quantified, normalized and pooled. Pooled libraries are bound to the surface of a flow cell and each bound template molecule is clonally amplified up to 1000-fold to create individual clusters. Four fluorescently labeled nucleotides are then flowed over the surface of the flow cell and incorporated into each nucleic acid chain. Each nucleotide label acts as a terminator for polymerization, thereby ensuring that a single base is added to each nascent chain during each cycle. Fluorescence is measured for each cluster during each cycle to identify the base that was added to each cluster. The dye is then enzymatically removed to allow incorporation of the next nucleotide during the next cycle.

The raw read counts were transformed using voom and quantile normalized, which results in expression measures (summarized intensities) in log base 2. Statistical analysis was subsequently performed using empirical Bayes from the limma R package. Normalized data provide the input for statistical hypothesis testing, in which genes are identified that are statistically significantly different between sample groups, i.e. differences that are unlikely to be due to chance. The degree of difference, i.e. the fold-change, is also accounted for. In the output, the fold-changes (logFC) are given as log2 values, with a positive logFC representing up-regulation, and a negative logFC indicating down-regulation. For each comparison (e.g. A-B, which is the same as 'A relative to B'), the first group (A) is the numerator, while the second group (B) is the denominator. Thus, a positive logFC for the comparison 'A-B' indicates up-regulation in A relative to B. Comparisons were undertaken using linear modelling. Subsequently, empirical Bayesian analysis was applied (including vertical *p*-value adjustment for multiple testing, which controls for false discovery rate). For each comparison, the null hypothesis was that there was no difference between the groups being compared. Significant genes with adjusted *P*<0.05 from each comparison were analyzed for enrichment of KEGG and GO pathway membership using a hypergeometric test. Enrichment (*P*<0.05) was assessed for up- and down-regulated genes separately. The results from the three separate xenograft models were taken and a congruence analysis was performed to determine the extent of overlap between the differentially expressed genes and significantly enriched KEGG pathways and GO terms between cell lines.

RNA-seq data is available on the NCBI GEO repository under accession number GSE83479.

### Sequencing of SS18-SSX translocation

25 mg frozen tissue was processed for RNA extraction using the RNeasy mini kit (QIAGEN) according to the manufacturer’s instructions. A cDNA pool was generated using SuperScript III First-Strand Synthesis SuperMix (Invitrogen) according to the manufacturer’s instructions. PCR was performed using the following primers:

PCR_SSX1_F ATTCCTCACAACATTACTACGAAGG

PCR_3UTR_SSX1_R TGACGAGGGAGCCGCAGCCA

PCR_SSX2_F AGGAGGAAATTCACAGTATGGCC

PCR_3UTR_SSX2_R ATGACGAGGGGTCCGCA

SYT-SSX1_F GAGGACGAAAATGATTCGAAG

SYT-SSX1_R GCTTTTCCTGGGGGGTGCAG

SYT-SSX2_F GAAGGAAATGATTCGGAGG

SYT-SSX2_R GGTTTTCCCGGGGGGCACAGC

The PCR products were analyzed on gel and gel purified prior to sequencing. Sequencing was performed using the BigDye Terminator Cycle Sequencing. Data analysis was performed with DNASTAR Lasergene software.

### ELISA

Histones were acid extracted as previously described [22] and H3K27Me3 ELISA on treated samples was performed as previously described [6]. Histone amounts loaded on the ELISA plates were 30 ng and 300 ng for H3 plate and H3K27Me3 plate, respectively. Histones were prepared in equivalent concentrations [0.3 ng/μL for H3 analysis and 3 ng/μL for H3K27Me3 analysis] in coating buffer (PBS with 0.05% BSA [Jackson ImmunoResearch]). Sample or standard protein (Active Motif) was coated on Immulon 4HBX plates (Thermo Scientific) overnight at 4°C. The following day, plates were washed three times with 300 μL per well PBST (PBS with 0.05% Tween 20; 10× PBST; KPL; 51-14-02). Plates were blocked with 300 μL per well of diluent [PBS plus 2% (wt/vol) BSA plus 0.05%Tween 20], incubated at room temperature for 2 h, and washed three times with PBST. All antibodies were diluted in diluent. One hundred microliters per well rabbit monoclonal anti-H3K27Me3 antibody (CST; 9733; 50% glycerol stock, 1:1,000) or rabbit polyclonal anti-total H3 antibody (Abcam; ab1791; 50% glycerol stock, 1:5,000) were added to each plate. Plates were incubated for 90 min at room temperature and washed three times with PBST. One hundred microliters per well horseradish peroxidase conjugated anti-rabbit IgG antibody (CST; 7074) was added at 1:2,000 to the H3K27Me3 plate and at 1:6,000 to the H3 plate and incubated for 90 min at room temperature. Plates were washed four times with PBST. For detection, 100 μL per well 3,3’,5,5’-tetramethylbenzidine substrate (BioFx Laboratories; TMBS) were added, and plates were incubated at room temperature for approximately 3 min. Reaction was stopped with 100 μL per well of 0.5 mol/L H_2_SO_4_. Subsequently, the developed colors in the wells were measured using a plate spectrophotometer (SpectraMax 250, Molecular Devices) at 450 nm (reference wavelength 650 nm).

### Quantitative real-time PCR (qPCR)

For *in vitro* samples, total RNA isolation and cDNA synthesis were performed using the TaqMan Gene Expression Cells-to-CT kit (Life technologies) according to the manufacturer's protocol. *ATF3*, *SOX2*, *EGR1*, *CDKN2A*, and *GAPDH* expression were analyzed by using the TaqMan Gene Expression Assays (Life technologies) and the TaqMan probes (Hs00231069_m1, Hs01053049_s1, Hs00152928_m1, Hs00233365_m1, and Hs99999905_m1, respectively). The expression levels were adjusted to *GAPDH* and expressed as fold changes to DMSO control. For *in vivo* samples, tumor samples were collected at approximately 3 hours after the last dose from mice treated with tazemetostat for 7 days (Fuji and HS-SY-II xenografts). The tumor samples were stored using RNAlater (Life technologies). Total RNA isolation and the reverse transcription were performed by RNeasy Mini Kit (Qiagen) and High capacity cDNA Reverse Transcription kit (Life technologies) according to the manufacturer’s protocol. The cDNA samples were used for real time-PCR using the TaqMan probes described above with additional housekeeping gene *18s rRNA* (Hs99999901_s1). The expression levels were adjusted to *GAPDH* and *18s rRNA*, and expressed as fold changes to vehicle control.

### Immunohistochemistry (IHC)

Collected tumors were subjected to formalin fixation, embedded in paraffin. Paraffin sections were prepared, deparaffinized and processed for IHC following a general process according to a polymer-based method (EnVision, DAKO). IHC for SMARCB1 and CD57 IHC, as well as SS18-SSX FISH was performed at PhenoPath from tumor samples prepared as described above. The scoring indicates the percentage of positive tumor cells and the modal intensity of staining in the positive cells on an intensity scale of 0–3 (0 = no staining [negative]; 1+ = weak staining; 2+ = moderate staining; and 3+ = strong staining).

### Karyotype Analysis

Karyotype analysis of the Fuji cells was performed at Department of Biological Sciences, Hokkaido University Graduate School of Science. Fifty independent metaphases were counted for karyotype analysis.

Karyotype and MBand analysis of the HS-SY-II cells was performed at Brigham and Women’s Hospital CytoGenomics Core Facility. Twelve independent metaphases were counted for karyotype analysis, with a chromosome count of 52–61 observed. Full chromosomal analysis is in Supporting Information. Twenty five independent MBands were analyzed.

## Results

### Synovial sarcoma cell lines are sensitive to EZH2 inhibition *in vitro*

**Fig 1:**
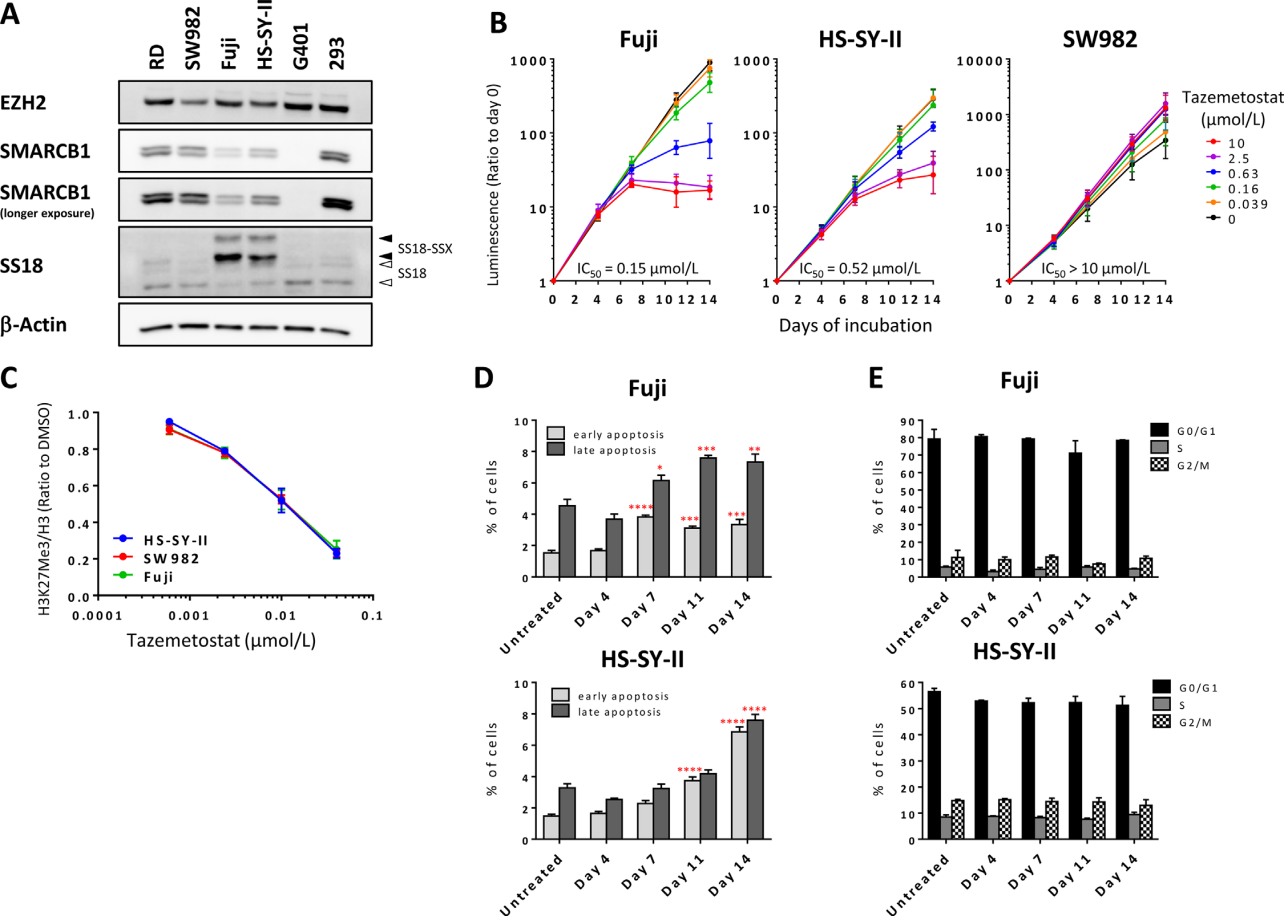
SS18-SSX translocation positive synovial sarcoma cell lines are sensitive to EZH2 inhibition *in vitro*. (A) Immunoblot analysis of human soft tissue sarcoma (RD and SW982), human synovial sarcoma (Fuji and HS-SY-II), human MRT (G401), and human embryonic kidney (293) cell lines to examine expression of EZH2, SMARCB1, SS18/SS18-SSX, and β-actin (loading control). Closed and open arrowheads represent SS18–SSX and SS18, respectively. (B) The synovial sarcoma cell lines were treated at increasing doses of tazemetostat for 14 days and proliferation was assessed at the indicated timepoints. Tazemetostat was refreshed on days 4 and 11. Data represent the geometric mean ± 95% confidence interval in triplicate from 3 independent experiments. IC_50_, 50% inhibitory concentration value. (C) H3K27Me3 levels were assessed in HS-SY-II, Fuji and SW982 treated with tazemetostat for 96 hours. Levels were quantified using ELISA and data is represented as the ratio of H3K27Me3 to total H3 and are shown relative to the DMSO control of each concentration. Data are represented as mean values ± SEM (n = 3). (D, E) Apoptosis analysis (Annexin V-FITC assay) (*D*) or cell-cycle analysis (by flow cytometry) (*E)* of Fuji and HS-SY-II treated with the IC_50_ for tazemetostat (0.15 μmol/L and 0.52 μmol/L, respectively). Data are represented as mean values ± SEM (n = 3). For panel D: * *P*< 0.05, ** *P*<0.01, *** *P*<0.001, **** *P*<0.0001, vs. untreated, one-way analysis of variance followed by the Dunnett’s multiple comparisons test.

Two synovial sarcoma cell lines (Fuji and HS-SY-II) were used for this study. As previously reported, the Fuji cell line contains an SS18-SSX2 translocation [23], while the HS-SY-II cell line a SS18-SSX1 translocation [24]; the RD and SW982 cell lines are soft tissue sarcoma cell lines that do not contain an SS18-SSX translocation [18] and were included as controls. Although the SW982 cell line was formerly classified as synovial sarcoma, the molecular hallmark of synovial sarcoma is the SS18-SSX translocation [17], and therefore the SW982 cell line is likely to be mis-classified and represent a separate, distinct type of soft tissue sarcoma. We confirmed the presence of a translocation in Fuji and HS-SY-II by immunoblot using an antibody for SS18, and the translocation was not detected in RD, SW982, nor in two additional non-synovial sarcoma cell lines included as negative controls (G401 and 293) (Fig 1A). Additionally, the absence of mutations in EZH2 in these two synovial sarcoma cell lines was confirmed by sequencing (S1A Fig). SMARCB1 expression was quantified by densitometry and normalized to control β-actin levels, and found to be decreased in the synovial sarcoma cell lines relative to other sarcoma cell lines (Fig 1A, S1B Fig). This is consistent with previous reports that suggest that decreased SMARCB1 is due to the expression of the SS18-SSX translocation [9, 25, 26]. The SMARCB1 deficiency observed in the synovial sarcoma cell lines could be distinguished from the SMARCB1-negative malignant rhabdoid tumor (MRT) cell line, G401 (Fig 1A). Altered levels of H3K27Me3 were not observed in any of the two synovial sarcoma cell lines (S1C Fig), in contrast with the WSU-DLCL2, an *EZH2*-mutant cell line, which we have previously shown displays very high levels of H3K27Me3 [27].

We have previously shown that SMARCB1-negative MRT tumors are dependent on EZH2 activity [6], and we therefore investigated whether the synovial sarcoma cell lines, with decreased SMARCB1 expression, would likewise display a similar sensitivity to EZH2 inhibition. Indeed, treatment of Fuji or HS-SY-II cell lines with the EZH2 inhibitor tazemetostat [6] (chemical structure is shown in S1D Fig) led to a concentration-dependent decrease in proliferation over a 14-day assay (IC_50_ values on day 14 were 0.15 μmol/L [95% CI: 0.065–0.35] and 0.52 μmol/L [95% CI: 0.36–0.75], respectively), while the translocation-negative SW982 cell line displayed no decrease in growth rate (IC_50_ values > 10 μmol/L) (Fig 1B). In the same assay, G401 also showed a concentration-dependent decrease in proliferation (S1E Fig) as we have previously shown [6]. These proliferation dependencies were not due to differential activity of tazemetostat inhibiting H3K27Me3, as the concentration-dependent decrease in H3K27Me3 observed in these three cell lines were indistinguishable from one another (Fig 1C). In line with the observed antiproliferative activity in 14-day proliferation assays we also detected a time-dependent induction of apoptosis in both Fuji and HS-SY-II cells when treated with the IC_50_ for tazemetostat (0.15 μmol/L and 0.52 μmol/L, respectively) for up to 14 days (Fig 1D and S1G Fig). Supporting the result, caspase-3/7 activity was also increased in tazemetostat-treated Fuji, whereas the increase was not obvious in HS-SY-II (S1F Fig). However, cell cycle analysis showed no change with the treatment in both cell lines (Fig 1E and S1G Fig), suggesting that the cell death observed are not in conjunction with cell cycle arrest. This suggests that SS18-SSX translocation containing synovial sarcoma cell lines are dependent on the activity of the PRC2 complex and EZH2 for their survival.

### EZH2 inhibition induces changes in synovial sarcoma gene expression

**Fig 2:**
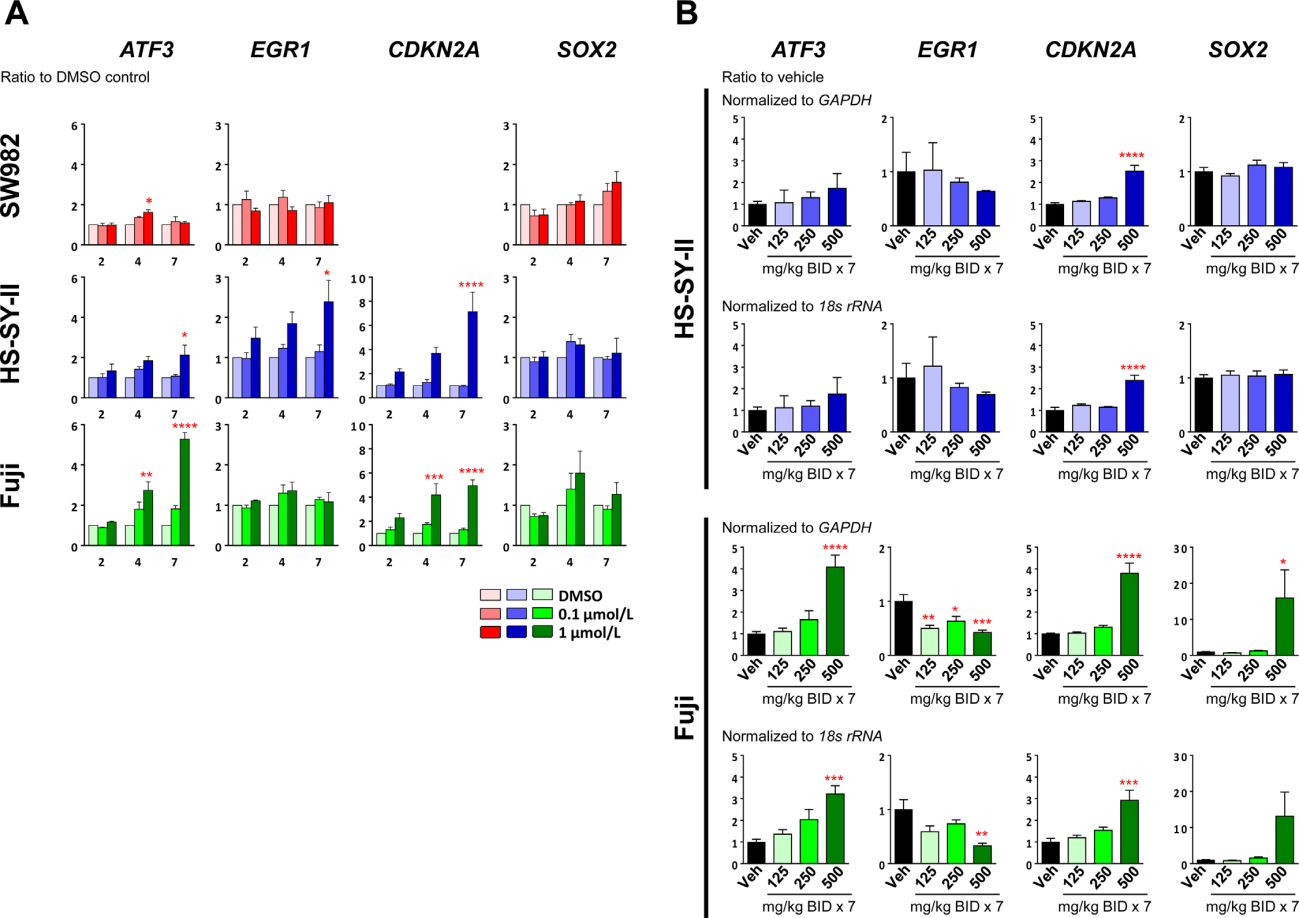
EZH2 inhibition modulates expression of synovial sarcoma-related genes. (A) Quantitative real-time PCR (qPCR) analysis of the indicated cell lines treated with tazemetostat at the indicated doses for 2, 4 or 7 days. Data are normalized to *GAPDH* expression relative to the vehicle control. Data are represented as mean values ± SEM (n = 3). (B) qPCR analysis of HS-SY-II or Fuji xenograft samples treated with tazemetostat at the indicated doses, twice daily, for 7 days. Tumors were harvested 3 hours after the last dose on day 7. Data are normalized to *GAPDH* expression or *18s rRNA* expression relative to the vehicle control and represents the average of 5 independent animals per group ± SEM. For this whole figure: * *P*< 0.05, ** *P*<0.01, *** *P*<0.001, **** *P*<0.0001, vs. vehicle control, one-way analysis of variance followed by the Dunnett’s multiple comparisons test.

Alterations in SWI/SNF and PRC2 activity is thought to induce tumorigenesis through altered epigenetic regulation of gene transcription [28]. Several pathways have been implicated in synovial sarcoma, including altered ATF2-mediated transcription, aberrant cell cycle inhibition and modulation of *SOX2* expression [9, 17, 23]. ATF2-target genes, specifically *ATF3* and *EGR1*, have been previously reported to be transcriptionally repressed by the SS18-SSX fusion protein via recruitment of repressive complexes (including PRC2) to ATF2 target genes [23], and these altered transcription profiles may be in part responsible for translocation-induced tumorigenesis. In agreement with this, we observed that inhibition of EZH2 by tazemetostat treatment led to an increase in *ATF3* mRNA levels in a concentration and time-dependent manner in both the Fuji and HS-SY-II cell lines, but not the SW982 cell line (Fig 2A). However, increased *EGR1* expression after tazemetostat treatment was not consistently observed across cell lines, as the HS-SY-II but not Fuji showed a dose and time-dependent increase in expression (Fig 2A). Additionally, we observed an increase in expression of the known PRC2 target gene and tumor suppressor, *CDKN2A* [3], with tazemetostat treatment, in both the Fuji and HS-SY-II cell lines (Fig 2A); the SW982 cell line has been previously shown to be deleted for *CDKN2A* [29]. Similar gene expression alterations were observed by the treatment with an alternative EZH2 inhibitor of similar potency, specificity and mode of action [N-((4,6-Dimethyl-2-oxo-1,2-dihydropyridin-3-yl)methyl)-3-(ethyl((*trans*)-4-((2methoxyethyl)(methyl)amino)cyclohexyl)-amino)-2-methyl-5-(3-morpholinoprop-1-yn-1-yl)benzamide, EPZ011989 [30], chemical structure is shown in S1D Fig] (S1H Fig).

A recent report suggests that a SS18-SSX fusion protein containing SWI/SNF complex in synovial sarcoma cell lines leads to displacement of PRC2/EZH2 at *SOX2* loci, resulting in loss of the repressive H3K27Me3 marks and increased *SOX2* expression [9]. We observed that EZH2 inhibition did not lead to a significant change in *SOX2* expression in any of the synovial sarcoma cell lines examined (Fig 2A). Thus, EZH2 inhibition does not change *SOX2* expression in SS18-SSX positive synovial sarcoma cell lines, but is able to reverse some synovial sarcoma-specific gene expression, at least *ATF3*, and others less consistently across models. The increased expression of the tumor suppressor gene *CDKN2A* possibly explains the proliferation and apoptosis effects observed in these cell lines.

We also examined the modulation of gene expression in the Fuji and HS-SY-II *in vivo* in xenograft models. Subcutaneous xenografts of the Fuji or HS-SY-II cell line were generated, and treated for 7 days with 125, 250 or 500 mg/kg tazemetostat twice daily (BID). The xenografts were then examined for expression changes in ATF2-target genes (*ATF3* and *EGR1*) and well as *CDKN2A* and *SOX2* (Fig 2B). Some similar changes to those seen in cell culture were observed, including increases in *ATF3* (in the Fuji xenograft) and *CDKN2A* expression (in both xenografts). However other changes differed from what was observed *in vitro*, including a lack of increase in EGR1 expression as well as an increase in *SOX2* expression only at the highest dose in the Fuji xenograft. Thus, EZH2 inhibition induces similar changes in gene expression *in vitro* and *in vivo*, although some context specific changes are observed.

### Anti-tumor activity is observed in a cell line xenograft model of synovial sarcoma

**Fig 3:**
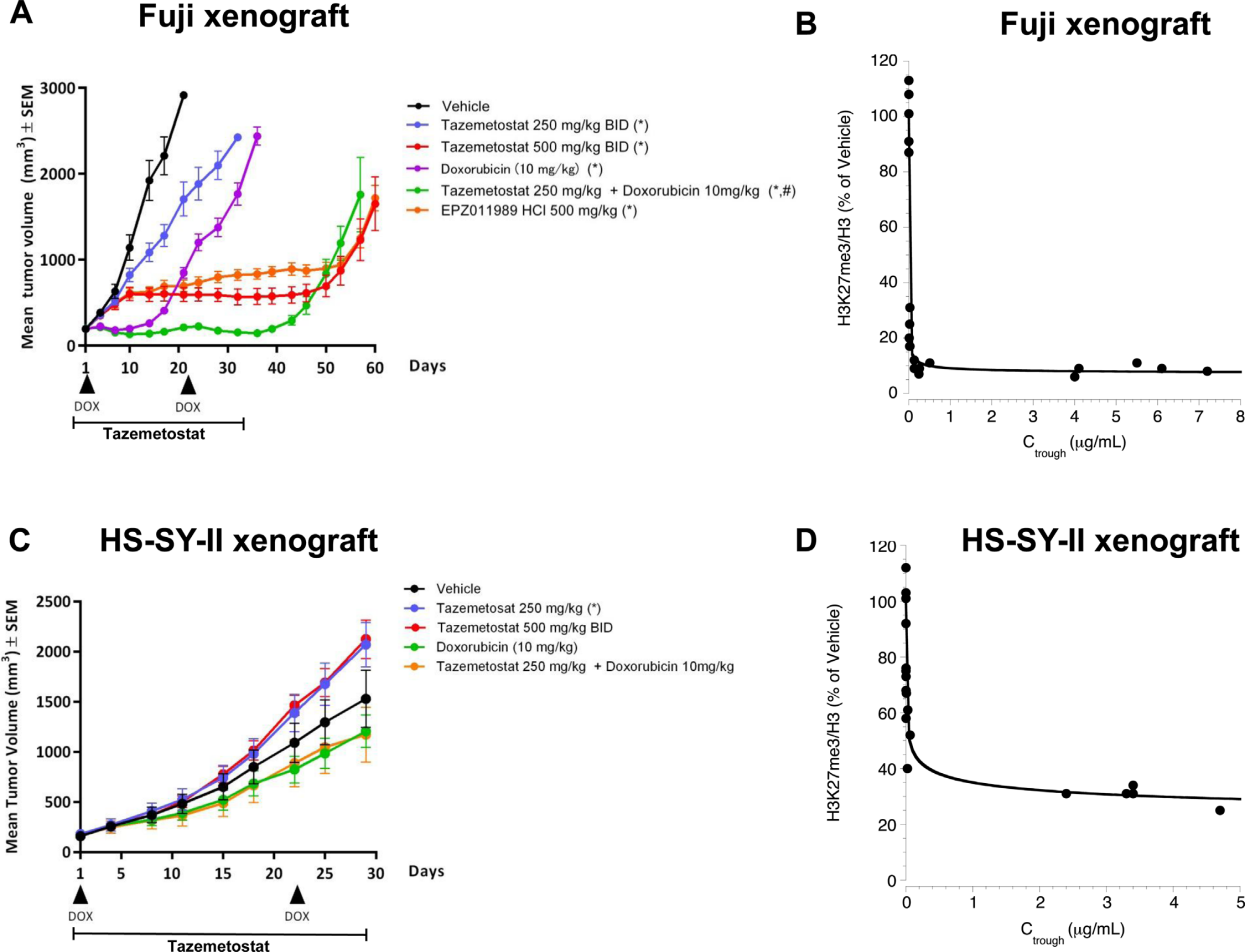
Effects of EZH2 inhibition in synovial sarcoma cell line xenograft models. (A) Tumor growth inhibition in Fuji xenograft induced by twice daily (BID) administration of tazemetostat for 35 days at the indicated dosage, with or without doxorubicin (10 mg/kg) treatment on days 1 and 22. An alternative EZH2 inhibitor, EPZ011989, was also included at 500 mg/kg BID. Treatment was stopped after 35 days and tumor regrowth was monitored. Data shown as mean values ±SEM; n = 7. Arrowheads indicate the administration of doxorubicin, lines indicate the dosing period for tazemetostat or EPZ011989. (B) EZH2 target inhibition in Fuji xenograft samples from mice treated with tazemetostat for seven days at the indicated doses in relationship to systemic C_trough_ levels of tazemetostat measured 5 minutes before the last dose on day 7. H3K27Me3 and H3 levels were measured in histones preparations by ELISA and data represents the ratio of H3K27Me3 to total H3. The horizontal line represents the mean. (C) Assessment of tumor growth in HS-SY-II xenograft model. Mice were treated with tazemetostat for 28 days at the indicated dosage, with or without doxorubicin (10 mg/kg) treatment on days 1 and 22. Data are shown as mean values ±SEM; n = 6 and representative of two independent experiments. Arrowheads indicate the administration of doxorubicin, horizontal arrows indicate the dosing period for tazemetostat. (D) EZH2 target inhibition in HS-SY-II xenograft samples from mice treated with tazemetostat for seven days at the indicated doses in relationship to systemic C_trough_ levels of tazemetostat measured 5 minutes before the last dose on day 7. H3K27Me3 and H3 levels were measured in histones preparations by ELISA and data represents the ratio of H3K27Me3 to total H3. The horizontal line represents the mean. * *P*<0.05 vs. vehicle, # *P*<0.05 vs. both 250 mg/kg tazemetostat and doxorubicin; one-way analysis of variance followed by Tukey’s multiple comparison test after logarithmic transformation.

To examine whether the growth inhibitory effects of EZH2 inhibition that we observed in cell culture were also present *in vivo*, we examined the effect of treatment with EZH2 inhibitors on Fuji and HS-SY-II xenografts. Studies were performed in Balb/C nude mice bearing subcutaneous Fuji or HS-SY-II xenografts. Mice were dosed orally for 35 days (Fuji xenograft) or 28 days (HS-SY-II xenograft) with either tazemetostat or EPZ011989. We included EPZ011989 (an alternative EZH2 inhibitor of similar potency, selectivity and mode of action [30]) as an additional control to demonstrate that effects on the xenografts are due to EZH2 inhibition and are not specific to tazemetostat exposure. Additionally, the effects of doxorubicin as a monotherapy or in combination with EZH2 inhibition were examined as doxorubicin is a standard of care for the treatment of patients with synovial sarcoma. Both tazemetostat and EPZ011989 alone were well tolerated with minimal effect on body weight; however, some body weight loss was observed in mice receiving doxorubicin (S2A Fig). In the fast growing Fuji xenograft model, treatment with 250 mg/kg or 500 mg/kg BID tazemetostat led to a dose-dependent decrease in tumor volume, and treatment with 500 mg/kg BID EPZ011989 also inhibited tumor growth (Fig 3A). The dose of 250 mg/kg BID tazemetostat induced tumor growth inhibition (43% at 14 days of treatment) and 500 mg/kg BID was sufficient to induce tumor stasis which continued until approximately 10 days after the cessation of dosing. The combination treatment with 250 mg/kg BID tazemetostat and doxorubicin showed superior antitumor effect compared to each monotherapy during the dosing period, although regrowth of the tumor was observed at approximately 8 days after cessation of dosing. A sub-cohort of mice (n = 5) were euthanized on day 7 to evaluate tazemetostat exposure and H3K27Me3 levels. The plasma levels of tazemetostat measured at either five minutes before or three hours after the final dose on day 7 revealed a dose-dependent increase in systemic exposure (S2B Fig), and H3K27Me3 levels were inhibited in an exposure-dependent manner (Fig 3B).

A similar study was carried out in the HS-SY-II xenografts, and as described for the Fuji xenograft study EZH2 inhibitor treatment was well tolerated (S2C Fig). In a parallel 7-day dosing study systemic exposure of tazemetostat was found to be dose-dependent and exposure–dependent decreases in intratumoral H3K27Me3 levels were observed (Fig 3D and S2D Fig). However, unlike in the Fuji xenograft model, EZH2 inhibition did not lead to a decrease in tumor growth (Fig 3C), although H3K27Me3 levels were found to be inhibited in tumors collected at study end (S3 Fig), and these cells were growth inhibited with tazemetostat *in vitro* (Fig 1B and 1D). To examine potential causes for these discrepancies in xenograft sensitivity, we investigated whether there may be genetic or phenotypic differences between these two models. Synovial sarcomas are almost always diploid tumors with a very low frequency of chromosomal aberrations and mutations other than the *SS18-SSX* translocation [17, 31–36]. The Fuji cell line was found to be a diploid cell line when first isolated, and we confirmed the chromosome number to be 45 (50 cells counted, data not shown), which was the same as previously reported [37]. In contract, the HS-SY-II karyotype has been described as a hypertriploid karyotype with many additional chromosomal alterations in addition to the SS18-SSX translocation [24]. Further examination in which twelve independent metaphases were counted confirmed a karyotype ranging from 52–61 chromosomes per cell, with many chromosomal alterations observed in addition to the SS18-SSX translocation, including a large number that were clonal in nature (S4A Fig). MBand analysis further found that the HS-SY-II cell line has variable numbers of t(x;18) fusions (mode number of 2) along with many other clonal and non-clonal chromosomal abnormalities involving chromosomes X and 18 (S4B Fig). This karyotype is suggestive of a cell line with a highly unstable genotype that is undergoing ongoing chromosomal changes, and thus may not be representative of typical synovial sarcomas. Additionally, this data shows that the HS-SY-II cell line is highly heterogeneous, and raises the possibility that the tumor cells that contribute to the xenograft growth *in vivo* may not be representative of the *in vitro* cell line, potentially explaining the differences in the phenotypic effects of EZH2 inhibition *in vitro* vs. *in vivo*. Expression of CD57/Leu-7, a marker which is highly expressed in many synovial sarcoma patients [38–41], displayed mixed expression in the original tumor from which HS-SY-II was isolated [24], suggestive of a mixed population of cells. It was previously reported [24] and we further confirmed that while the HS-SY-II *in vitro* cell line has high levels of CD57, the expression of CD57 is significantly lower in the xenograft (S4C Fig). Thus the *in vitro* and *in vivo* models for HS-SY-II may represent highly distinct cell populations. Lastly, we examined expression of SMARCB1, which shows an altered expression pattern in the majority of synovial sarcomas [25, 42]. While the levels of SMARCB1 were indeed low in both the Fuji and HS-SY-II cell lines *in vitro* (Fig 1A), only the Fuji xenograft displayed a decrease in SMARCB1 staining while the HS-SY-II xenograft displayed no such decrease (S4D Fig). As the deficiency in SMARCB1 expression was the original hypothesis that led us to investigate the use of EZH2 inhibitors in synovial sarcoma, this may explain the differential sensitivity observed in this model. As such, we believe that the karyotype, unstable genotype, CD57 staining, and lack of SMARCB1-deficiency suggest that HS-SY-II is not a representative xenograft for SMARCB1-deficient synovial sarcoma.

### Anti-tumor activity is observed in two PDX models of synovial sarcoma

**Fig 4:**
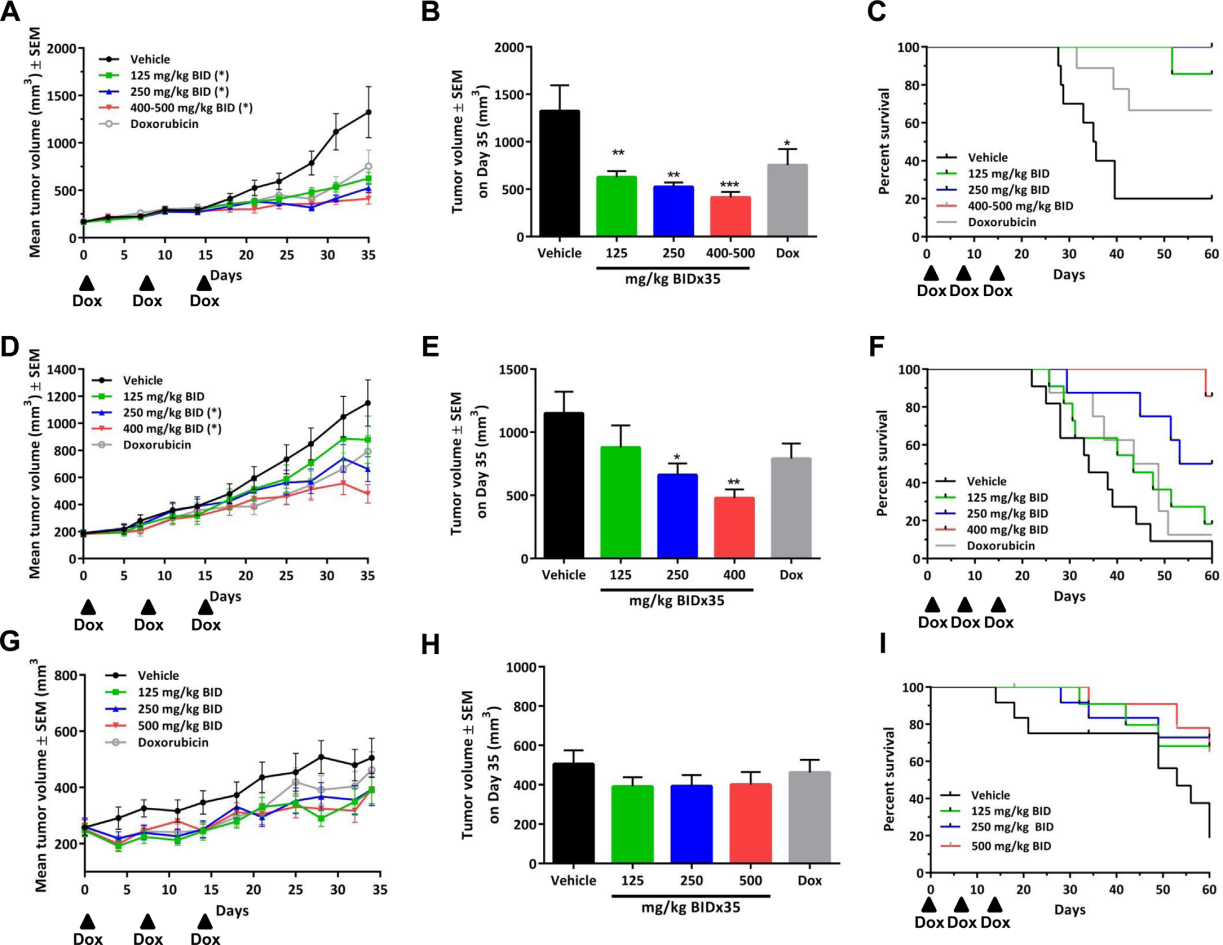
Tazemetostat treatment inhibits growth of synovial sarcoma patient-derived xenograft (PDX) models. Three individual synovial sarcoma PDX models were dosed with tazemetostat on a BID schedule for 35 days at the indicated doses: CTG-0771 (A-C), CTG-0331 (D-F), and CTG-1169 (G-I). Arrowheads indicate the administration of doxorubicin, horizontal arrows indicate the dosing period for tazemetostat. For the CTG-0771 model tazemetostat was dosed at 500 mg/kg from days 1–17 followed by 400 mg/kg from days 19–35. A control group in each study was dosed with doxorubicin on days 1, 7, and 14. (A, D, G) Tazemetostat induced significant tumor growth inhibition in two of three synovial sarcoma PDX models. Data shown as mean values ±SEM; n = 12 per model. * *P*<0.05 vs. vehicle group, two-tailed one-way analysis of variance followed by the Dunnett’s multiple comparisons test. (B, E, H) Mean tumor volumes ±SEM on at the end of dosing (day 35) are presented. * *P*<0.05, ** *P*<0.01, *** *P*<0.001 vs. vehicle group, one-way analysis of variance followed by the Dunnett’s multiple comparisons test. (C, F, I) For the PDX, models 3 to 5 mice per group with the largest tumors were euthanized on day 35 for blood and tissue sampling, and the 7 remaining mice were followed for an additional 25 days with biweekly tumor measurements. Kaplan-Meier plots show that tazemetostat increased the survival of mice in a dose-dependent manner for CTG-0331 and CTG-0771, as assessed by a tumor volume endpoint of 1200 mm^3^. Note that in panel C the lines for the 250 and 400–500 mg/kg groups are overlapping. Kaplan-Meier plot for CTG-1169 was assessed by a tumor volume endpoint of 600 mm^3^ (a smaller endpoint was used due to the slow growing nature of this model).

Given the importance of using model systems that most accurately recapitulate the clinical disease, we also investigated how synovial sarcoma PDX models respond to EZH2 inhibition *in vivo*. Three PDX models in NCr nude mice, with tumors implanted unilaterally in the flank region with tumor fragments, were used for this study. The *SS18-SSX* translocation was confirmed in these models both by fluorescent *in situ* hybridization (FISH) (S5A Fig) as well as sequencing of the *SS18-SSX* translocation (S5B Fig). The tumors were treated BID for 35 days with 125 or 250 mg/kg tazemetostat or weekly with 3 mg/kg doxorubicin as a control. An additional higher dose of tazemetostat (400 or 500 mg/kg) was also included. Minimal changes in body weight were observed (S5D Fig), with the exception of the 500 mg/kg dose of tazemetostat. This led us to decrease the dose to 400 mg/kg in one of the models at day 19 and proceed with 400 mg/kg BID for one of the other models. Significant tumor growth inhibition in response to tazemetostat treatment was observed in two of the three models; in the third model there was a trend to tumor growth inhibition albeit not statistically significant (Fig 4A–4I and S5C Fig). This tumor growth inhibition was observed to be dose-dependent for one of the models, CTG-0331 (Fig 4D–4F and S5C Fig), while in the CTG-0771 model all doses of tazemetostat induced significant tumor growth inhibition (Fig 4A–4C and S5C Fig), and in the slow growing CTG-1169 model only a modest decrease in growth was observed (Fig 4G–4I and S5C Fig). For all PDX models five mice per group with the largest tumors were euthanized 3 hours after the last dose on day 35 for blood and tissue sampling, and the 7 remaining mice were followed for an additional 25 days with biweekly tumor measurements. Mice treated with tazemetostat experienced a dose-dependent tumor growth delay when defining a tumor volume endpoint of 1200 mm^3^ (Fig 4C and 4F), with the CTG-0771 model showing the strongest effect. No significant tumor growth delay was observed for the CTG-1169 model when defining a tumor volume endpoint of 600 mm^3^ for this slower growing model (Fig 4I and S5C Fig). As in the xenograft models, a dose dependent increase in tazemetostat exposure and inhibition of intratumoral H3K27Me3 was observed (S5E and S5F Fig). We additionally examined SMARCB1 expression levels in the PDX models. As would be expected, all three models showed a decrease in SMARCB1 staining in the tumor relative to the endothelium (S5G Fig). Interestingly, the model which showed the greatest sensitivity to EZH2 inhibition (CTG-0771) was also the model that displayed the greatest decrease in SMARCB1 staining. Taken together, dependence on EZH2 was observed in synovial sarcoma models *in vitro*, one xenograft model *in vivo*, as well as two of three PDX models *in vivo*.

To further examine the gene expression patterns induced by EZH2 inhibition, we performed RNA-seq analysis on the three *in vivo* models that were sensitive to EZH2 inhibition (CTG-0331, CTG-0771, and Fuji). The gene expression changes measured by qPCR (described in Fig 2) were confirmed with this data set as well (data not shown). For the Fuji model, we examined samples for all tazemetostat doses at day 35, and for the PDX models we examined only samples from mice treated at the highest dose at day 35. The Fuji model displayed dose- and time-dependent increases in gene expression changes (S5H Fig). At day 35, all three models displayed significant overlap in the affected gene pathways by KEGG and GO pathways analysis, including decreases in pathways associated with metabolism and cell cycle, and increases in pathways associated with apoptosis and cell death processes (S5I and S5J Fig, overlapping gene pathways listed in S1 Table). Thus, synovial sarcoma *in vivo* models display overlapping EZH2 inhibitor-induced changes in gene expression, suggesting a common mechanism for the antitumor activity of EZH2 inhibition.

## Discussion

In this report, we investigate the potential that synovial sarcomas, due to their deficiency in SMARCB1, may be dependent on EZH2 activity. Indeed, we find that *in vitro*, *SS18-SSX* translocation-positive synovial sarcoma cell lines are sensitive to the EZH2 inhibitor tazemetostat. Furthermore, these cell lines display tazemetostat-induced changes in gene expression consistent with modulation of genes aberrantly expressed in synovial sarcoma that include well known targets of PRC2, such as cell cycle regulators. We additionally observed sensitivity to EZH2 inhibition in a SMARCB1-deficient synovial sarcoma xenograft, but not a synovial sarcoma xenograft expressing wild-type levels of SMARCB1. Furthermore, two PDX models of synovial sarcoma displayed sensitivity to EZH2 inhibition. A third PDX model tested showed only a modest and not statistically significant tumor growth inhibition when compared to vehicle. This model, however, displayed very slow growth characteristics which could in part explain the more subtle effect of tazemetostat dosing during the study observation period. Thus, SMARCB1-deficient synovial sarcoma represents an additional indication which may display clinical response to EZH2 inhibition. While this manuscript was under review an additional study using a structurally similar inhibitor of EZH2 also reported sensitivity to EZH2 inhibition in cell culture models of synovial sarcoma [43] including additional cell line models. Our study expands on this work by examining *in vivo* models as well as gene expression changes observed both *in vitro* and *in vivo*.

We have previously shown that SMARCB1-negative MRT tumors are sensitive to EZH2 inhibition *in vitro* and *in vivo* [6]. In addition, Italiano and colleagues recently reported evidence of antitumor activity in tazemetostat-treated adult patients with SMARCB1-negative MRT [8]. In MRT, SMARCB1 is lost due to biallelic inactivation of its gene, whereas in synovial sarcoma, SMARCB1 levels are lowered due to the presence of the SS18-SSX fusion protein. This suggests that SMARCB1 expression can be reduced by multiple mechanisms, potentially leading to sensitivity in other cancer types. Interestingly, the HS-SY-II model is sensitive *in vitro* but not *in vivo*, correlating with a lack of SMARCB1 loss in the *in vivo* model. Additionally, of the three PDX models, the most sensitive model displays the greatest decrease in SMARCB1 expression. It has been reported that clinically 86% of synovial sarcoma tumors display low SMARCB1 expression, although the degree of expression varies across tumor specimens [25, 26]. Further studies are needed to explore the relationship between SMARCB1 expression and EZH2 dependence in synovial sarcoma. Furthermore, other members of the SWI/SNF complex are frequently lost or mutated in cancers [4, 28], and it will be of interest to investigate whether altered SWI/SNF activity through mutation or expression changes of a given complex subunit in general predicts sensitivity to EZH2 inhibition.

The mechanism(s) by which *SS18-SSX* translocations lead to tumorigenesis is currently being investigated by different groups, with at least two mechanisms currently suggested. Two reports from Nielsen and colleagues [23, 44] have suggested that the oncogenic SS18-SSX fusion protein leads to recruitment of the PRC2 complex to ATF2 target genes, resulting in aberrant inhibition of the transcription of these target genes. ATF2 target genes include *ATF3* and *EGR1*, which regulate proliferation, stress response, differentiation and apoptosis, among other functions. In agreement with this, we see that treatment with tazemetostat does lead to an increase in *ATF3* expression in the *SS18-SSX* positive but not negative synovial sarcoma cell lines. Modulation of *EGR1* expression, however, was less consistent. This suggests that different ATF2 target genes may be differentially impacted by either the SS18-SSX fusion protein and/or EZH2 inhibition. There also may be cell line specific differences, such as basal expression level of the target genes, expression of *ATF2*, or SS18-SSX expression. And finally, gene expression changes may be different *in vitro* and *in vivo*, highlighting the need to examine multiple markers under both conditions.

Another mechanism that has been suggested for SS18-SSX induced tumorigenesis is via assembly of aberrant SWI/SNF complexes which are abnormally targeted, specifically to the *SOX2* loci [9]. This increased binding at the *SOX2* loci results in a decrease in H3K27Me3 levels at the promoter, suggesting that the aberrant SWI/SNF complex displaces PRC2/EZH2 leading to the loss of repressive chromatin marks. However, we observe no increase in *SOX2* expression upon treatment with EZH2 inhibitors *in vitro*. This suggests either that PRC2 function at the *SOX2* loci is already fully inhibited in translocation positive synovial sarcoma cells, and thus *SOX2* expression cannot be upregulated any further, or that other mechanisms are regulating *SOX2* expression. However, an increase in *SOX2* expression was observed in the Fuji xenograft at the highest dose, a dose at which tumor stasis was observed. Thus, the observed antiproliferative effect of EZH2 inhibition is likely a result of the balance between multiple different pathways (proliferation, survival, etc.). In support of this, we observed modulation of other pathways that would predict sensitivity to EZH2 inhibition, including increased *CDKN2A*, which is both a known target of PRC2/EZH2 as well as a tumor suppressor. Of note, we do observe upregulation of *SOX2* in a MRT cell line (G401) treated with EZH2 inhibition (unpublished data); however, the cell line displays exquisite sensitivity to inhibition of EZH2 and display modulation of other genes which may override the changes in *SOX2* expression. These data suggest that *SOX2* expression alone does not dictate the proliferation response, at least in MRT. Thus, in determining the phenotypic outcomes of PRC2 inhibition it will be important to monitor the expression of multiple pathways and not just the expression of one gene.

In summary, the data presented in this work suggest that synovial sarcomas, like MRTs, are SMARCB1-deficient tumors that are sensitive to EZH2 inhibition. These observations warrant further investigations in a clinical trial setting. Correlative translational studies in conjunction with these clinical trials will be important to decipher whether other genetic or transcriptomic factors besides the presence of the oncogenic SS18-SSX fusion protein will influence sensitivity to EZH2 inhibition.

## Supporting Information

S1 Fig Further characterization of synovial sarcoma cell lines. (A) Sequencing analysis of *EZH2* hot spots revealed no mutations in either the Fuji or HS-SY-II cell line. (B) Immunoblot results from Fig 1A were quantitated by densitometry and normalized to control β-actin levels. Data are represented as mean values ± SEM (n = 3). (C) Immunoblot analysis of 293, G401, HS-SY-II, SW982, Fuji, WSU-DLCL2 and OCI-LY19 cell lines to examine levels of H3K27Me3, H3K27Me2 and total H3. (D) Chemical structure of tazemetostat and EPZ011989. (E) G401 cell line was treated at increasing doses of tazemetostat for 14 days and proliferation was assessed at the indicated time points. Tazemetostat was refreshed on days 4 and 11. Data represent the mean in triplicate from 2 independent experiments. (F) Apoptosis analysis (Caspase 3/7 assay) of Fuji and HS-SY-II treated with each IC_50_ of tazemetostat, 0.15 μmol/L and 0.52 μmol/L, respectively. Data are represented as mean values ± SEM (n = 3). ** *P*<0.01, *** *P*<0.001, vs. untreated, one-way analysis of variance followed by the Dunnett’s multiple comparisons test. (G) Flow data of panels 1D and 1E. (H) qPCR analysis of two synovial sarcoma (Fuji and HS-SY-II) and soft tissue sarcoma (SW982) cell lines treated with EPZ011989 at the indicated doses for 2, 4 or 7 days. Data are normalized to *GAPDH* expression relative to the vehicle control. Data are represented as mean values ± SEM (n = 3). * *P*< 0.05, ** *P*<0.01, *** *P*<0.001, **** *P*<0.0001, vs. vehicle control, one-way analysis of variance followed by the Dunnett’s multiple comparisons test.
(PDF)

S2 Fig Body weight and systemic exposures in synovial sarcoma cell line xenograft models. (A) Body weight measurements from mice dosed as in Fig 3A (Fuji xenograft). (B) Plasma levels of mice treated as in panel A with tazemetostat for 7 days on a twice daily (BID) schedule. The horizontal lines represent the mean. Open circles are plasma samples collected 5 minutes before the last dose, and filled circles samples collect 3 hours after the final dose. (C) Body weight measurements from mice dosed as in Fig 3C (HS-SY-II xenograft). (D) Plasma levels of mice treated as in panel C with tazemetostat for 7 days on a BID schedule. The horizontal lines represent the mean. Open circles are plasma samples collected 5 minutes before the last dose, and filled circles samples collected 3 hours after the final dose.
(PDF)

S3 Fig H3K27Me3 inhibition in HS-SY-II model after 28 days of dosing. Mice from the HS-SY-II xenograft study (Fig 3C) were dosed as indicated for 28 days on a twice daily (BID schedule). Histones from tumors collected at study end were subjected to ELISAs with antibodies specific to H3K27Me3 and H3. Data represent the ratio of H3K27Me3 to total H3. The horizontal lines represent the mean.
(PDF)

S4 Fig The HS-SY-II cell line and xenograft are not representative of SMARCB1-deficient synovial sarcomas. (A) Karyotype analysis of HS-SY-II cell line. *Top*: The karyotype observed across 12 individual cells; the number in the brackets represents the number of cells the chromosomal abnormality was observed in out of 12. *Bottom*: Representative karyotype analysis of one cell from the HS-SY-II cell line. Arrows in bold indicate clonal structural rearrangements; non-bolded arrows indicate non-clonal structural rearrangements; and red arrows indicate the translocation between chromosome 18 and X. (B) Representative MBand analysis of HS-SY-II cell line. Analysis of chromosome X probes (*left*) and chromosome 18 probes (*right*) reveal multiple abnormalities and translocations involving these two chromosomes. Below each analysis are the karyotype information and how frequency the aberration was observed in a group of 25 cells examined in total. At the bottom is the probe analysis from a cell line with no abnormalities in chromosome X or 18. (C) Quantification of CD57 expression by immunohistochemistry (IHC) in the HS-SY-II cell line *in vitro* and from a xenograft. The horizontal line represents the mean. Quantification was performed using a scale from 0 to 3. (D) *Top*: Quantification of SMARCB1 staining by IHC analysis in Fuji and HS-SY-II cell line xenografts in tumor and endothelium. The horizontal line represents the mean. Quantification is performed on a scale from 0 to 3. *Bottom*: Representative images from Fuji and HS-SY-II xenografts analyzed for SMARCB1 staining by IHC.
(PDF)

S5 Fig Tazemetostat treatment inhibits growth of synovial sarcoma patient derived xenograft (PDX) models. (A) FISH analysis of synovial sarcoma PDX models demonstrates the presence of the *SS18-SSX* translocation in all three models. (B) Sequencing of synovial sarcoma PDX models demonstrates the presence of the *SS18-SSX* translocation in all three models, and defines *SSX1* (CTG-1169) versus *SSX2* (CTG-0331 and CTG-0771) as the translocation partner. (C) Longitudinal tumor volume data for all twelve individual mice from the PDX studies presented in Fig 4. (D) Body weight measurements from mice dosed as indicated in Fig 4. (E) Plasma concentration of tazemetostat from mice treated for 35 days as indicated in Fig 4. The horizontal lines represent the mean. Pre samples are plasma samples collected 5 minutes before the last dose, and post samples are plasma samples collected 3 hours after the final dose on day 35. (F) EZH2 target inhibition in PDX models presented in Fig 4. H3K27Me3 and H3 levels were measured by ELISA and data represent the ratio of H3K27Me3 to total H3. (G) *Top*: Quantification of SMARCB1 staining by IHC analysis in the PDX models in the tumor and endothelium. The horizontal line represents the mean. Quantification is performed on a scale from 0 to 3. *Bottom*: Representative images for SMARCB1 staining by IHC. (H) Analysis of RNA-seq data from the Fuji xenograft. Volcano plots comparing each tazemetostat dose at day 7 or day 35, showing the dose and time-dependent increase in gene expression changes. Significant up-regulated are shown in red and down-regulated are shown in blue. (I and J) Venn diagram showing overlap in gene expression changes by GO (*I*) and KEGG (*J*) analysis. Upregulated signatures are in the left panels and downregulated in the right panel. The list of GO and KEGG pathways which are overlapping in panel I or J are listed in S1 Table.
(PDF)

S1 Table Congruent GO Pathways Upregulated for CTG‐0331, CTG‐0771 and Fuji 250mg/kg. (PDF)
