# Correction: Preclinical Evidence of Anti-Tumor Activity Induced by EZH2 Inhibition in Human Models of Synovial Sarcoma

**DOI:** 10.1371/journal.pone.0170539

**Published:** 2017-01-13

**Authors:** Satoshi Kawano, Alexandra R. Grassian, Masumi Tsuda, Sarah K. Knutson, Natalie M. Warholic, Galina Kuznetsov, Shanqin Xu, Yonghong Xiao, Roy M. Pollock, Jesse J. Smith, Kevin W. Kuntz, Scott Ribich, Yukinori Minoshima, Junji Matsui, Robert A. Copeland, Shinya Tanaka, Heike Keilhack

The middle initial for two authors, Jesse Smith and Kevin Kuntz, are incorrect. Their names should read Jesse J. Smith and Kevin W. Kuntz respectively.

## References

[1] Kawano S, Grassian AR, Tsuda M, Knutson SK, Warholic NM, Kuznetsov G, et al (2016) Preclinical Evidence of Anti-Tumor Activity Induced by EZH2 Inhibition in Human Models of Synovial Sarcoma. PLoS ONE 11(7): e0158888 doi: 10.1371/journal.pone.0158888 27391784 10.1371/journal.pone.0158888 PMC4938529

