# Supplementary material for: Preclinical Evidence of Anti-Tumor Activity Induced by EZH2 Inhibition in Human Models of Synovial Sarcoma
Source: PLoS One. 2016 Jul 8;11(7):e0158888. doi: 10.1371/journal.pone.0158888 (PMC4938529; doi:10.1371/journal.pone.0158888)
Supplement: S1 Fig — (A) Sequencing analysis of EZH2 hot spots revealed no mutations in either the Fuji or HS-SY-II cell line. (B) Immunoblot results from Fig 1A were quantitated by densitometry and normalized to control β-actin levels. Data are represented as mean values ± SEM (n = 3). (C) Immunoblot analysis of 293, G401, HS-SY-II, SW982, Fuji, WSU-DLCL2 and OCI-LY19 cell lines to examine levels of H3K27Me3, H3K27Me2 and total H3. (D) Chemical structure of tazemetostat and EPZ011989. (E) G401 cell line was treated at increasing doses of tazemetostat for 14 days and proliferation was assessed at the indicated time points. Tazemetostat was refreshed on days 4 and 11. Data represent the mean in triplicate from 2 independent experiments. (F) Apoptosis analysis (Caspase 3/7 assay) of Fuji and HS-SY-II treated with each IC50 of tazemetostat, 0.15 μmol/L and 0.52 μmol/L, respectively. Data are represented as mean values ± SEM (n = 3). ** P<0.01, *** P<0.001, vs. untreated, one-way analysis of variance followed by the Dunnett’s multiple comparisons test. (G) Flow data of panels 1D and 1E. (H) qPCR analysis of two synovial sarcoma (Fuji and HS-SY-II) and soft tissue sarcoma (SW982) cell lines treated with EPZ011989 at the indicated doses for 2, 4 or 7 days. Data are normalized to GAPDH expression relative to the vehicle control. Data are represented as mean values ± SEM (n = 3). * P< 0.05, ** P<0.01, *** P<0.001, **** P<0.0001, vs. vehicle control, one-way analysis of variance followed by the Dunnett’s multiple comparisons test. (PDF) [file pone.0158888.s001.pdf]

S1 Fig

A

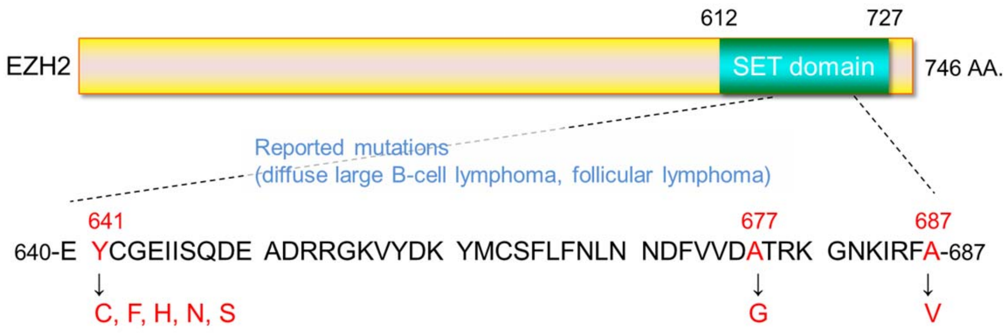

Cell line	Mutation in 640-687 AA. of EZH2
Fuji	No mutation
HS-SYII	No mutation

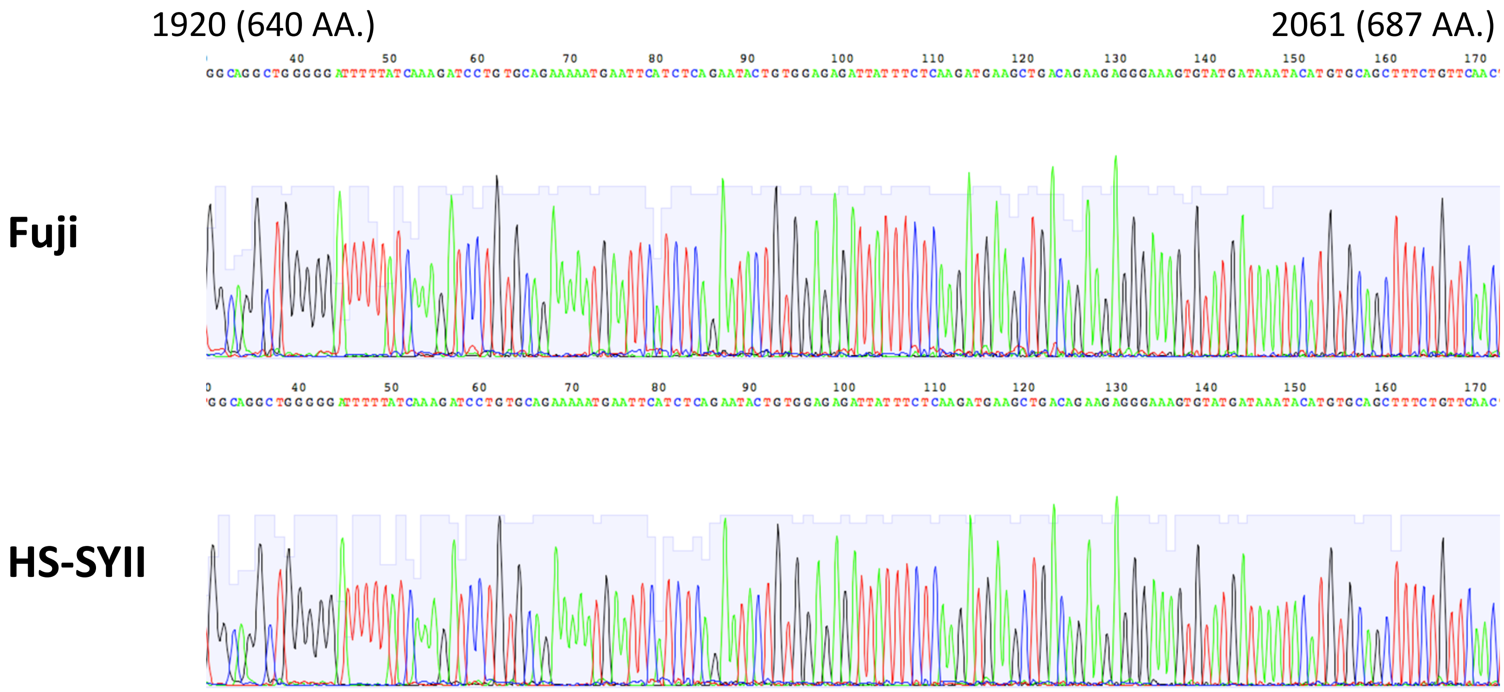

S1 Fig

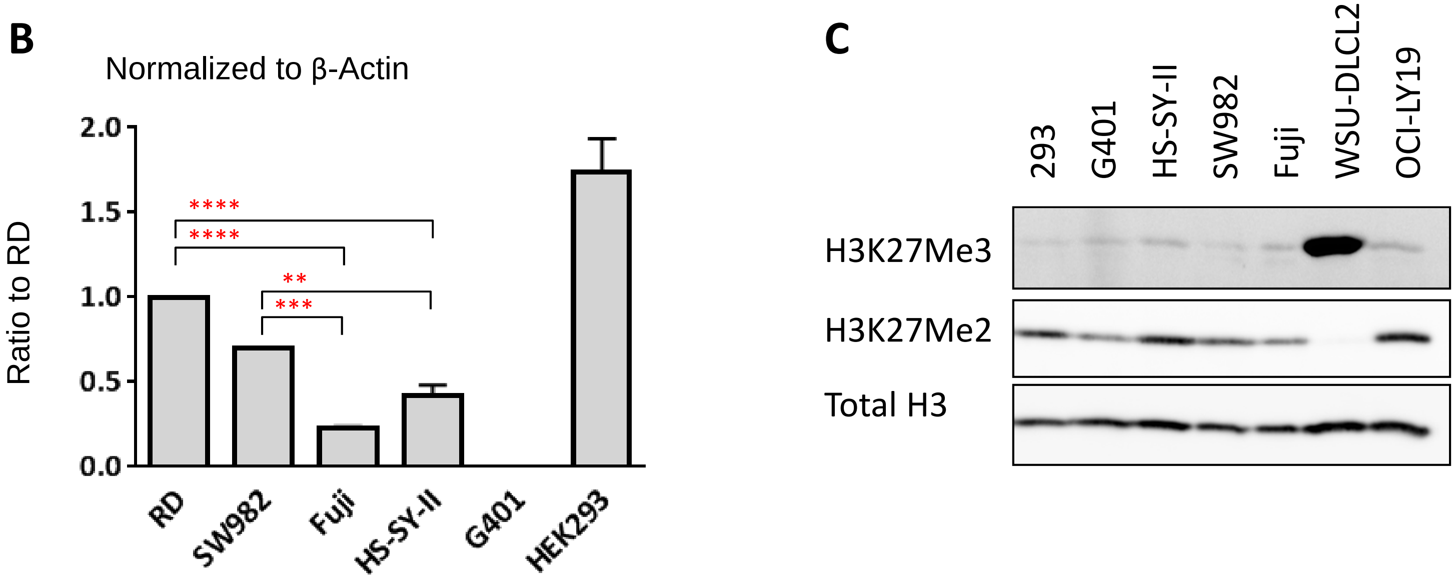
B
Normalized to β-Actin
Ratio to RD
2.0
1.5
1.0
0.5
0.0
****
****
**
***
RD
SW982
Fuji
HS-SY-II
G401
HEK293
C
293
G401
HS-SY-II
SW982
Fuji
WSU-DLCL2
OCI-LY19
H3K27Me3
H3K27Me2
Total H3

S1 Fig

D **Tazemetostat**

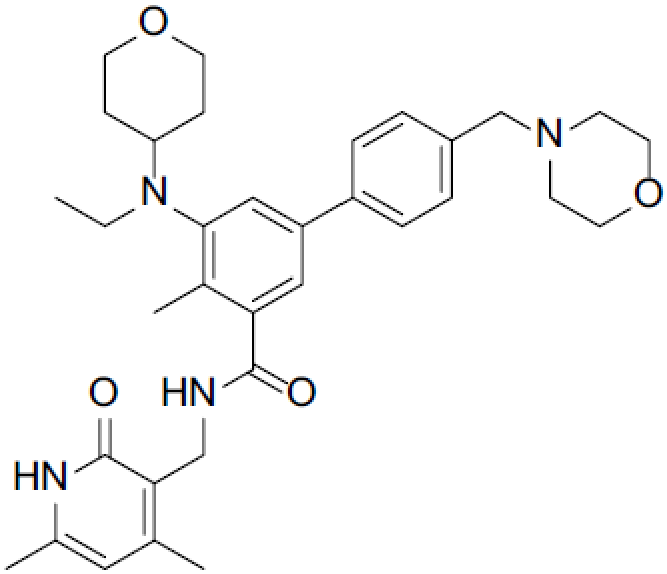

EPZ011989

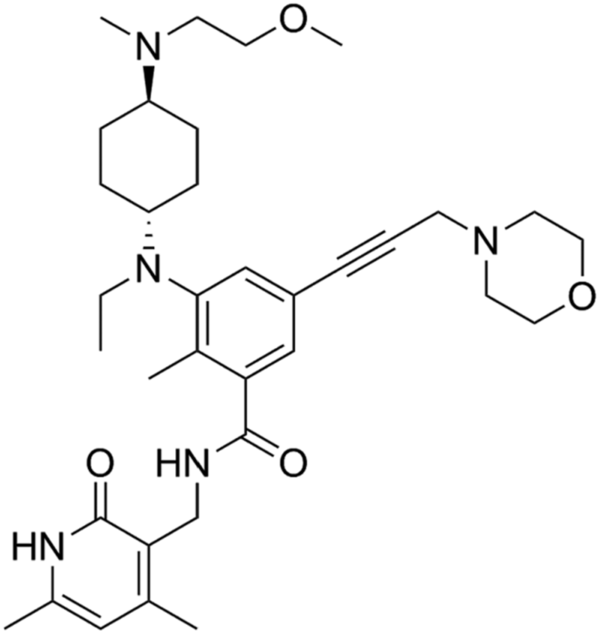

S1 Fig

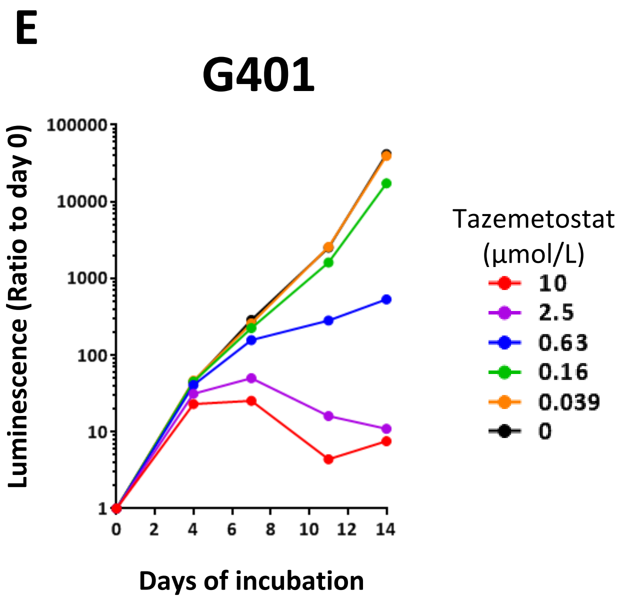
E
G401
Luminescence (Ratio to day 0)
100000
10000
1000
100
10
1
0
2
4
6
8
10
12
14
Days of incubation
Tazemetostat
(µmol/L)
10
2.5
0.63
0.16
0.039
0

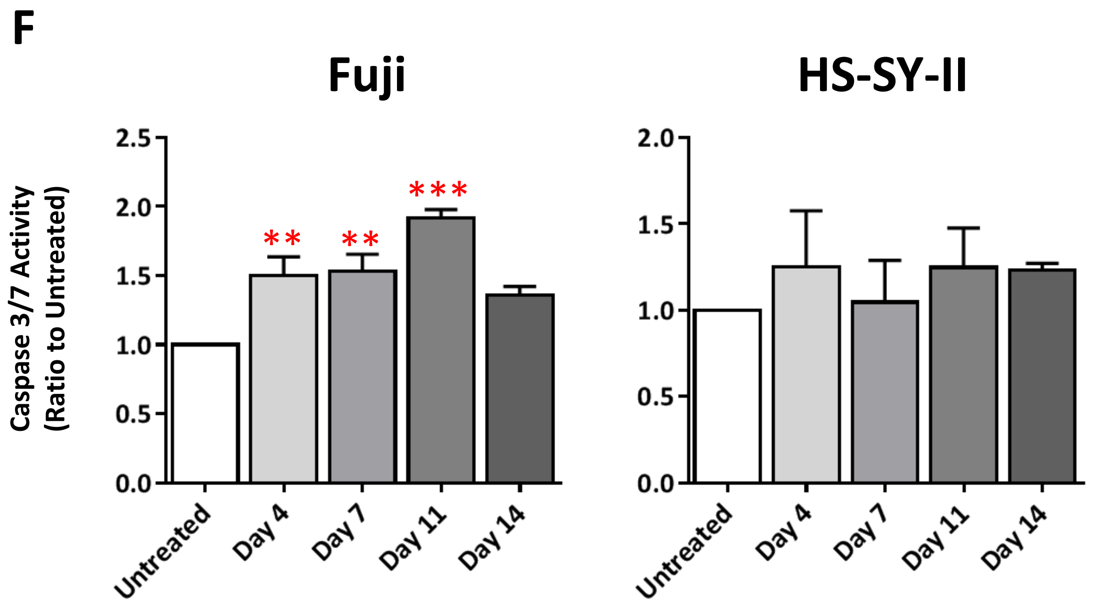
F
Fuji
HS-SY-II
Caspase 3/7 Activity
(Ratio to Untreated)
2.5
2.0
1.5
1.0
0.5
0.0
**
**
***
Untreated
Day 4
Day 7
Day 11
Day 14

S1 Fig

G

Apoptosis assay

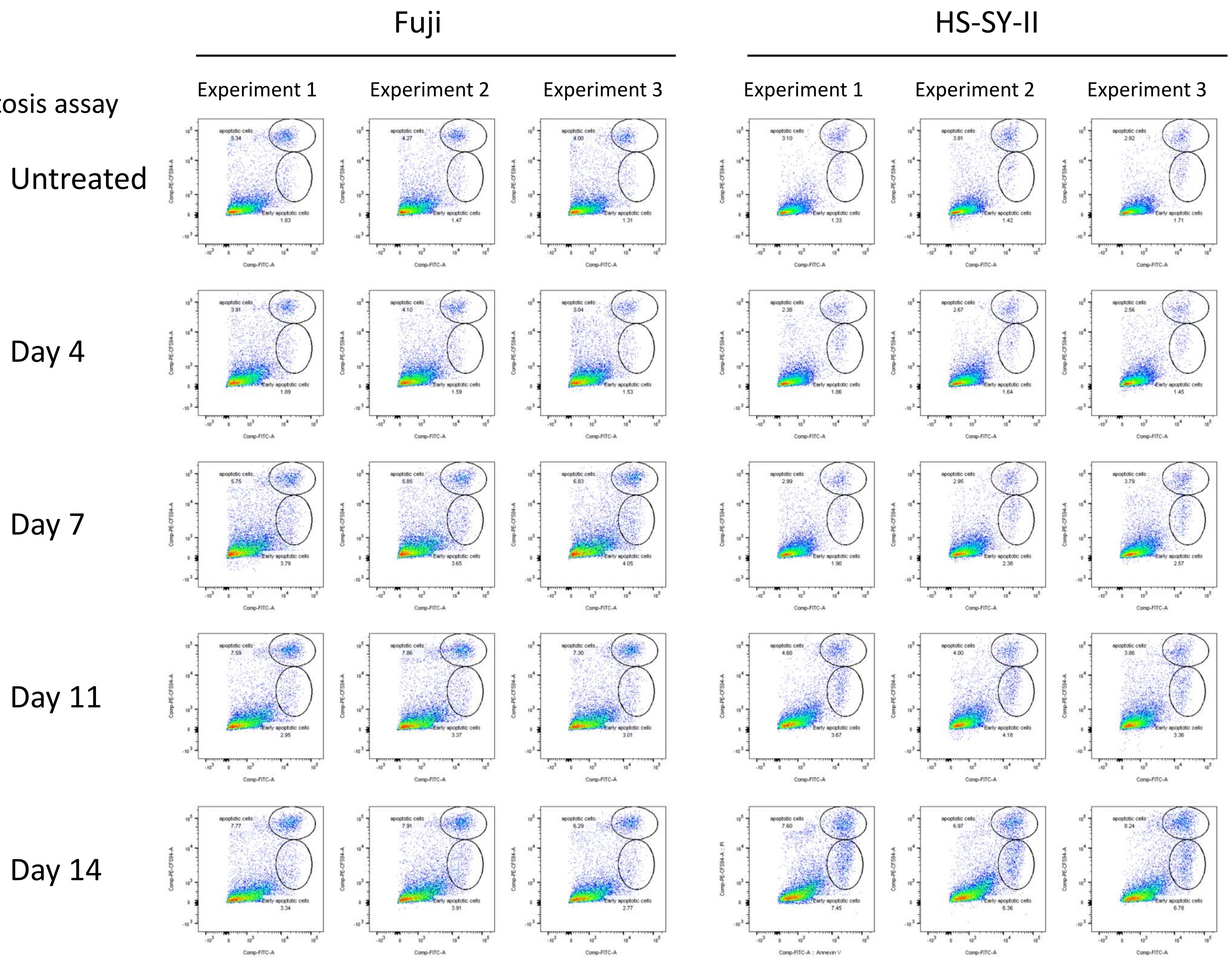

S1 Fig

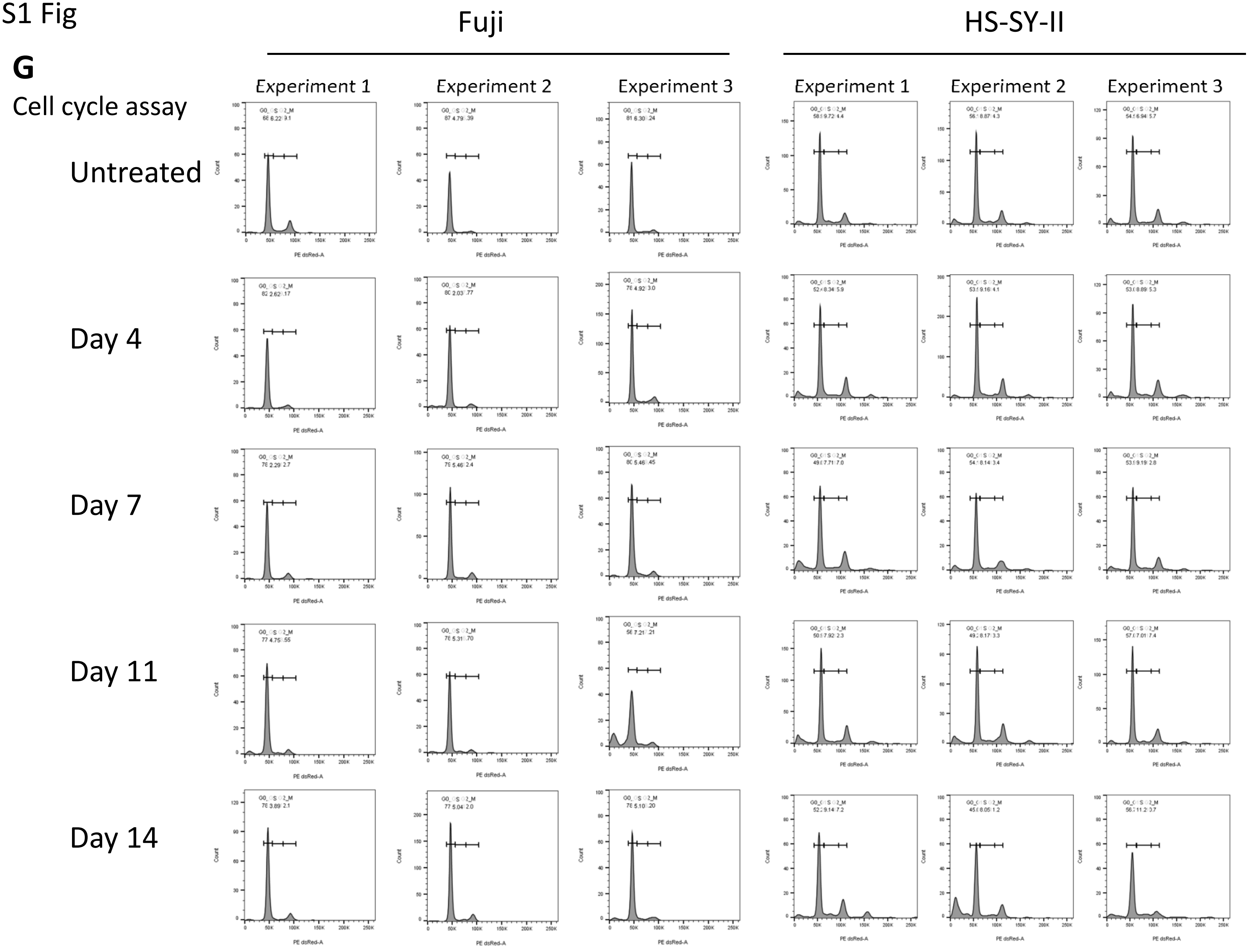
G
Cell cycle assay
Fuji
HS-SY-II
Experiment 1
Experiment 2
Experiment 3
Untreated
Day 4
Day 7
Day 11
Day 14
Count
PE dsRed-A

S1H Fig

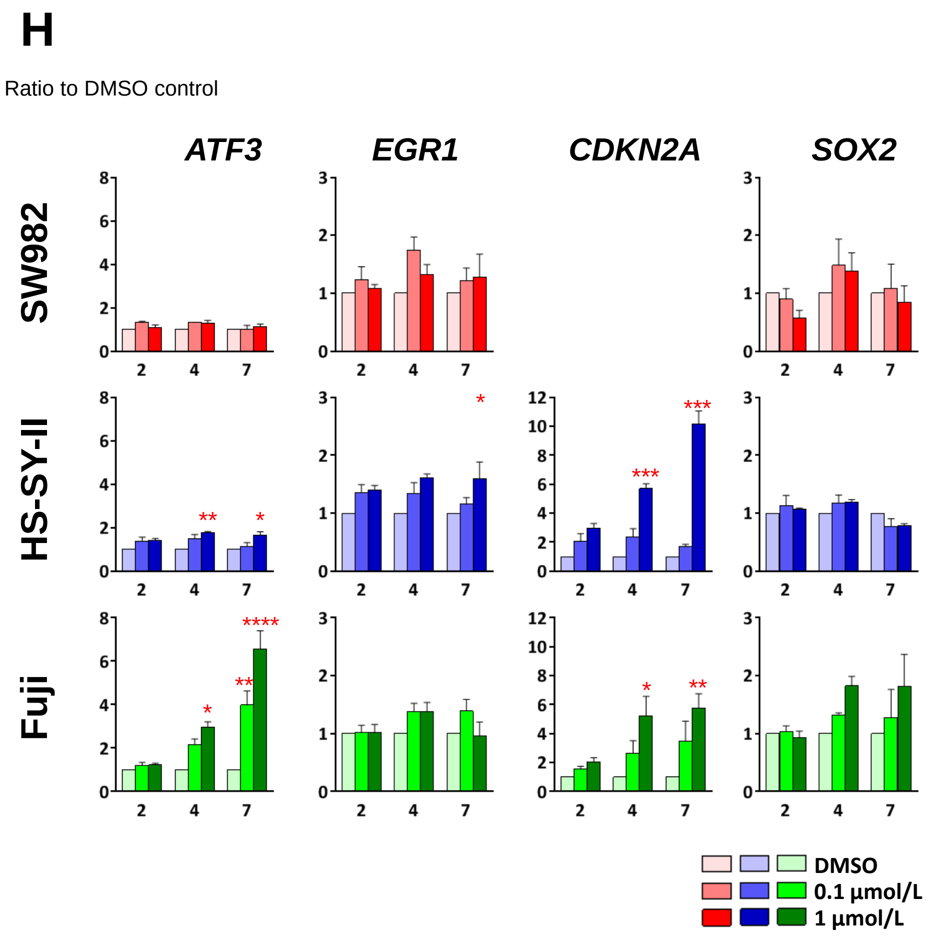
H
Ratio to DMSO control
ATF3
EGR1
CDKN2A
SOX2
SW982
HS-SY-II
Fuji
DMSO
0.1 µmol/L
1 µmol/L
