# Supplementary figures and images for: Preclinical Evidence of Anti-Tumor Activity Induced by EZH2 Inhibition in Human Models of Synovial Sarcoma
Source: PLoS One. 2016 Jul 8;11(7):e0158888. doi: 10.1371/journal.pone.0158888 (PMC4938529; doi:10.1371/journal.pone.0158888)

S2 Fig

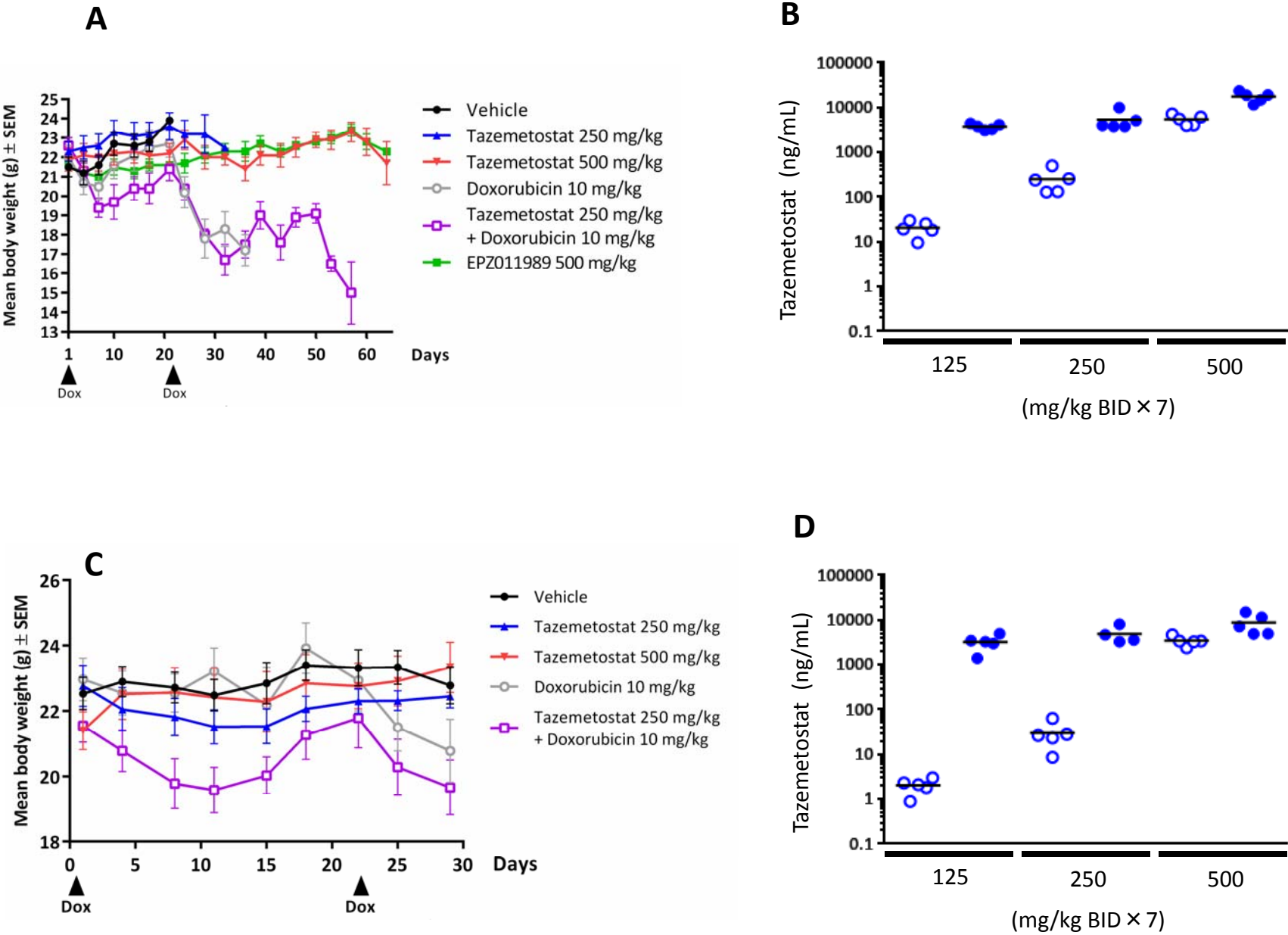

Supplement: S2 Fig — (A) Body weight measurements from mice dosed as in Fig 3A (Fuji xenograft). (B) Plasma levels of mice treated as in panel A with tazemetostat for 7 days on a twice daily (BID) schedule. The horizontal lines represent the mean. Open circles are plasma samples collected 5 minutes before the last dose, and filled circles samples collect 3 hours after the final dose. (C) Body weight measurements from mice dosed as in Fig 3C (HS-SY-II xenograft). (D) Plasma levels of mice treated as in panel C with tazemetostat for 7 days on a BID schedule. The horizontal lines represent the mean. Open circles are plasma samples collected 5 minutes before the last dose, and filled circles samples collected 3 hours after the final dose. (PDF) [file pone.0158888.s002.pdf]

S3 Fig

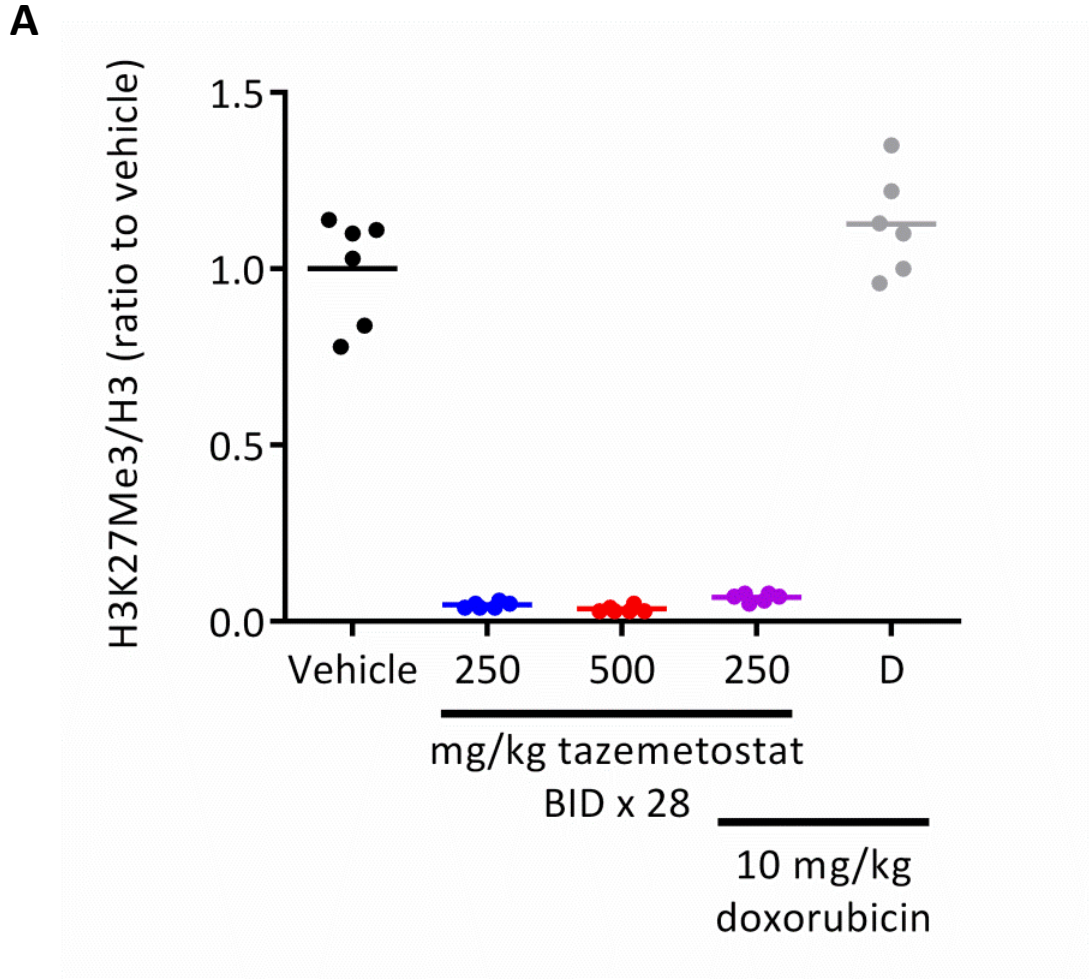

Supplement: S3 Fig — Mice from the HS-SY-II xenograft study (Fig 3C) were dosed as indicated for 28 days on a twice daily (BID schedule). Histones from tumors collected at study end were subjected to ELISAs with antibodies specific to H3K27Me3 and H3. Data represent the ratio of H3K27Me3 to total H3. The horizontal lines represent the mean. (PDF) [file pone.0158888.s003.pdf]
