# Supplementary material for: Preclinical Evidence of Anti-Tumor Activity Induced by EZH2 Inhibition in Human Models of Synovial Sarcoma
Source: PLoS One. 2016 Jul 8;11(7):e0158888. doi: 10.1371/journal.pone.0158888 (PMC4938529; doi:10.1371/journal.pone.0158888)

S4A Fig

A ***Karyotype of HS-SY-II:***

52~61<2n>,t(X;18)(p11.2;q11.2)x2[12],add(1)(q11)[9],+del(1)(p13)[7],add(2)(p23)[9],add(3)(p12)[2],del(4)(p13)[2],add(5)(q21)[5],add(5)(q31)[9],+der(5)t(1;5)(q33;q12)[12],+add(6)(q13)[11],+del(6)(p21.3)[6],+add(7)(p11)[12],+add(7)(q11.1)[6],del(8)(p21)[10],+add(8)(q11.2)[8],+9[6],+10[3],+add(12)(q13)[10], -13[8],add(13)(p11)[2],der(15)t(15;15)(p13;q11.2)[11],+16[10],-18[5],add(19)(q13.4)[9],-20[3],-21[3],22[12],del(22)(q11.2)[7],+mar1[4],+3-10mar[cp12]

Number in brackets represents number of clones in which rearrangement was observed out of 12.

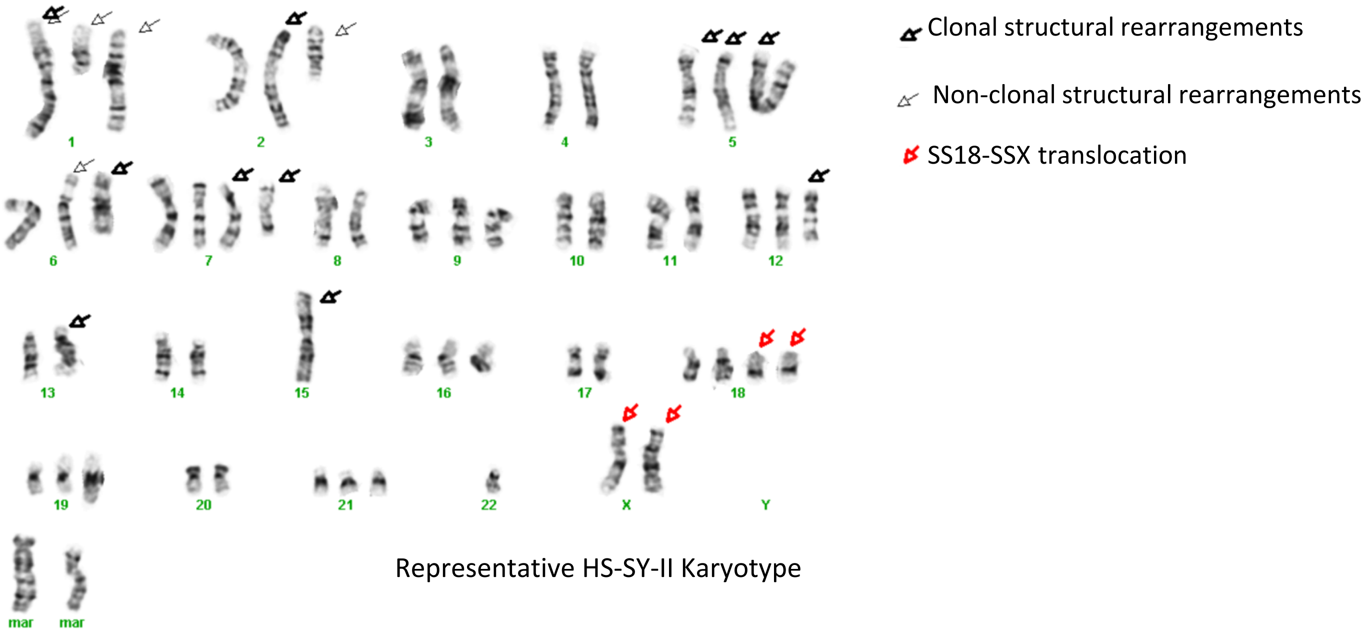

Representative HS-SY-II Karyotype

S4B Fig

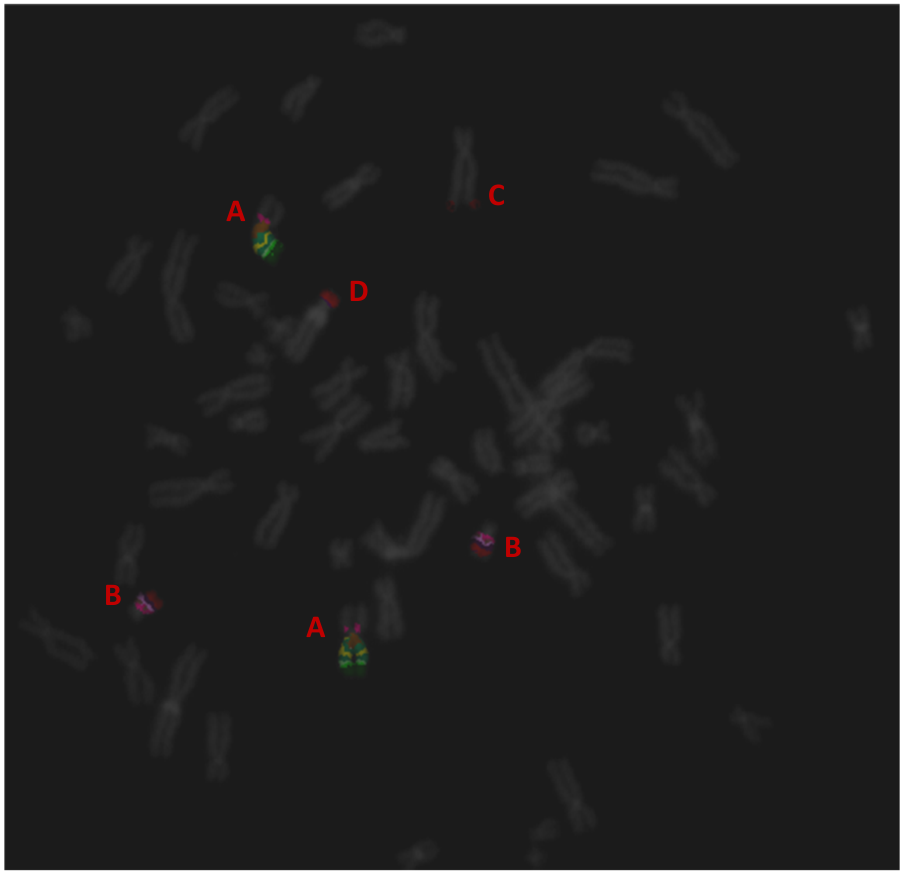

Metaphase spread 25: **X** MBAND

A=der(X)t(X;18) x2

B=der(18)t(X;18) x2

C=der(?)t(X;?) x1 (present in all cells)

D=different der(?)t(X;?) x1 (in 2cells)

(No normal X)

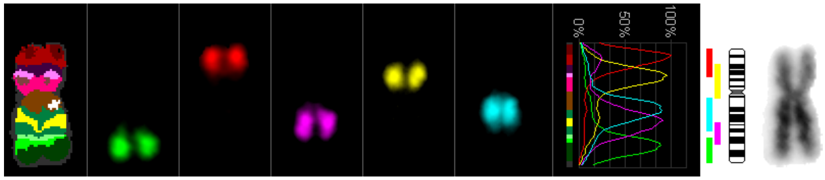

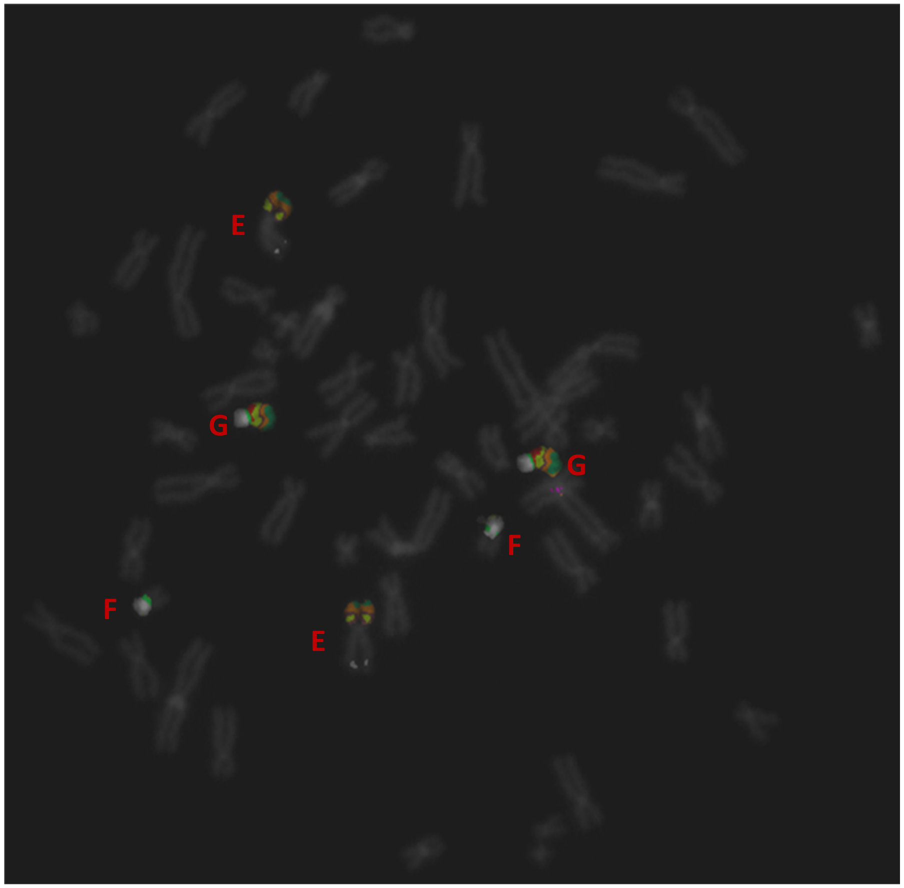

Metaphase spread 25a: **18** MBAND

E=der(X)t(X;18) x2

(note the small region on the opposite end which classifies as 18pter-q11; not consistently seen)

F=der(18)t(X;18) x2

G= normal 18 x2

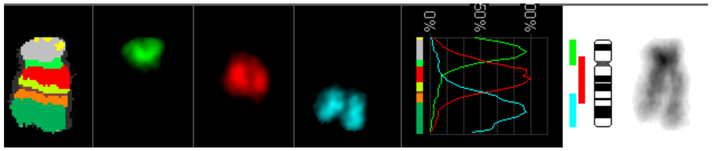

S4C and S4D Figs

C

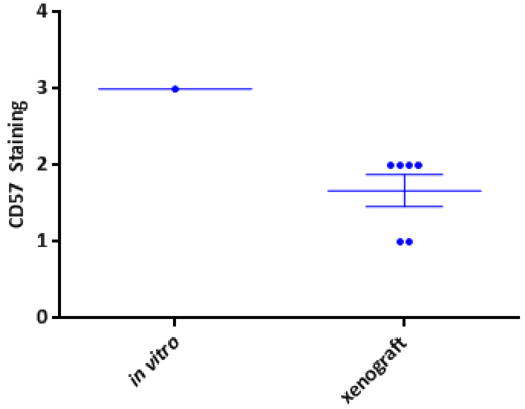

D

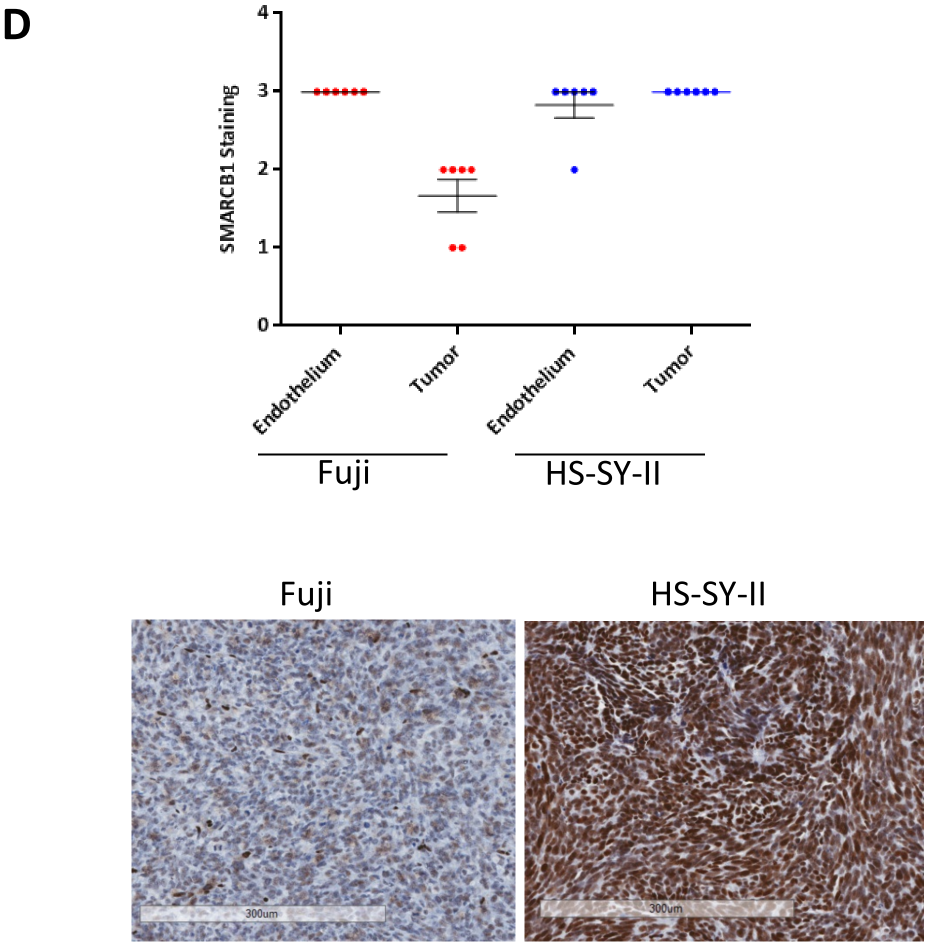

Supplement: S4 Fig — (A) Karyotype analysis of HS-SY-II cell line. Top: The karyotype observed across 12 individual cells; the number in the brackets represents the number of cells the chromosomal abnormality was observed in out of 12. Bottom: Representative karyotype analysis of one cell from the HS-SY-II cell line. Arrows in bold indicate clonal structural rearrangements; non-bolded arrows indicate non-clonal structural rearrangements; and red arrows indicate the translocation between chromosome 18 and X. (B) Representative MBand analysis of HS-SY-II cell line. Analysis of chromosome X probes (left) and chromosome 18 probes (right) reveal multiple abnormalities and translocations involving these two chromosomes. Below each analysis are the karyotype information and how frequency the aberration was observed in a group of 25 cells examined in total. At the bottom is the probe analysis from a cell line with no abnormalities in chromosome X or 18. (C) Quantification of CD57 expression by immunohistochemistry (IHC) in the HS-SY-II cell line in vitro and from a xenograft. The horizontal line represents the mean. Quantification was performed using a scale from 0 to 3. (D) Top: Quantification of SMARCB1 staining by IHC analysis in Fuji and HS-SY-II cell line xenografts in tumor and endothelium. The horizontal line represents the mean. Quantification is performed on a scale from 0 to 3. Bottom: Representative images from Fuji and HS-SY-II xenografts analyzed for SMARCB1 staining by IHC. (PDF) [file pone.0158888.s004.pdf]
