# Supplementary material for: Preclinical Evidence of Anti-Tumor Activity Induced by EZH2 Inhibition in Human Models of Synovial Sarcoma
Source: PLoS One. 2016 Jul 8;11(7):e0158888. doi: 10.1371/journal.pone.0158888 (PMC4938529; doi:10.1371/journal.pone.0158888)
Supplement: S5 Fig — (A) FISH analysis of synovial sarcoma PDX models demonstrates the presence of the SS18-SSX translocation in all three models. (B) Sequencing of synovial sarcoma PDX models demonstrates the presence of the SS18-SSX translocation in all three models, and defines SSX1 (CTG-1169) versus SSX2 (CTG-0331 and CTG-0771) as the translocation partner. (C) Longitudinal tumor volume data for all twelve individual mice from the PDX studies presented in Fig 4. (D) Body weight measurements from mice dosed as indicated in Fig 4. (E) Plasma concentration of tazemetostat from mice treated for 35 days as indicated in Fig 4. The horizontal lines represent the mean. Pre samples are plasma samples collected 5 minutes before the last dose, and post samples are plasma samples collected 3 hours after the final dose on day 35. (F) EZH2 target inhibition in PDX models presented in Fig 4. H3K27Me3 and H3 levels were measured by ELISA and data represent the ratio of H3K27Me3 to total H3. (G) Top: Quantification of SMARCB1 staining by IHC analysis in the PDX models in the tumor and endothelium. The horizontal line represents the mean. Quantification is performed on a scale from 0 to 3. Bottom: Representative images for SMARCB1 staining by IHC. (H) Analysis of RNA-seq data from the Fuji xenograft. Volcano plots comparing each tazemetostat dose at day 7 or day 35, showing the dose and time-dependent increase in gene expression changes. Significant up-regulated are shown in red and down-regulated are shown in blue. (I and J) Venn diagram showing overlap in gene expression changes by GO (I) and KEGG (J) analysis. Upregulated signatures are in the left panels and downregulated in the right panel. The list of GO and KEGG pathways which are overlapping in panel I or J are listed in S1 Table. (PDF) [file pone.0158888.s005.pdf]

S5A and S5B Figs

A

CTG-0331 CTG-0771 CTG-1169

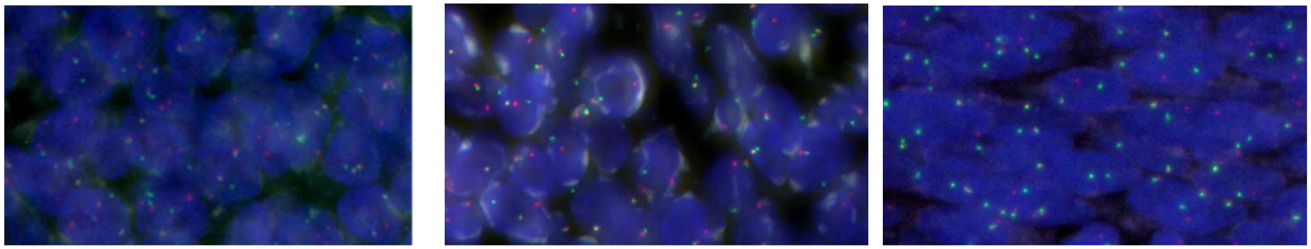

B

Observed Consensus Sequence for sample CTG-0331 (SYT-SSX2):

GGAGGAAATTCACAGTATGGCCAACAGCAAGATGCATACCAGGGACCACCTCCACAACAGGGATATCCACCCCAGCAGCAGCAGTACCCAGGGCAGCAAGGTTACCCAGGACAGCAGCAGGGCTACGGTCCTTCAC
AGGGTGGTCCAGGTCCTCAGTATCCTAACTACCCACAGGGACAAGGTCAGCAGTATGGAGGATATAGACCAACACAGCCTGGACCACCACAGCCACCCCAGCAGAGGCCTTATGGATATGACCAGatcatgcccaagaag
ccagcagaggaaggaaatgattcggaggaagtgccagaagcatctggcccacaaaatgatgggaaagagctgtgcccccgggaaaaccaactacctctgagaagattcacgagagatctggacccaaaagggggaacatgcctggacccacagactgcgtgagagaaaacag
ctggtgatttatgaagagatcagcgaccctgaggaagatgacgagtaa**CTCCCCTCAGGGATACGACACATGCCCATGATGAGAAGCAGAACGTGGTGACCTTTCACGAACATGGGCATGGCTGCGGACCCCTCGTCA**

Observed Consensus Sequence for sample CTG-0771 (SYT-SSX2):

AGGAGGAAATTCACAGTATGGCCAACAGCAAGATGCATACCAGGGACCACCTCCACAACAGGGATATCCACCCCAGCAGCAGCAGTACCCAGGGCAGCAAGGTTACCCAGGACAGCAGCAGGGCTACGGTCCTTCA
CAGGGTGGTCCAGGTCCTCAGTATCCTAACTACCCACAGGGACAAGGTCAGCAGTATGGAGGATATAGACCAACACAGCCTGGACCACCACAGCCACCCCAGCAGAGGCCTTATGGATATGACCAGatcatgcccaagaa
gccagcagaggaaggaaatgattcggaggaagtgccagaagcatctggcccacaaaatgatgggaaagagctgtgcccccgggaaaaccaactacctctgagaagattcacgagagatctggacccaaaagggggaacatgcctggacccacagactgcgtgagagaaaaca
gctggtgatttatgaagagatcagcgaccctgaggaagatgacgagtaa**CTCCCCTCAGGGATACGACACATGCCCATGATGAGAAGCAGAACGTGGTGACCTTTCACGAACATGGGCATGGCTGCGGACCCCTCGTCAT**

Observed Consensus Sequence for sample CTG-1169 (SYT-SSX1):

AGGAGGAAATTCACAGTATGGCCaACAGCAAGATGCATACCAGGGACCACCTCCACAACAGGGATATCCACCCCAGCAGCAGCAGTACCCAGGGCAGCAAGGTTACCCAGGACAGCAGCAGGGCTACGGTCCTTCA
CAGGGTGGTCCAGGTCCTCAGTATCCTAACTACCCACAGGGACAAGGTCAGCAGTATGGAGGATATAGACCAACACAGCCTGGACCACCACAGCCACCCCAGCAGAGGCCTTATGGATATGACCAGatcatgcccaagaa
gccagcagaggacgaaaatgattcgaagggagtgtcagaagcatctggcccacaaaacgatgggaaacaactgcaccccccaggaaaagcaaatatttctgagaagattaataagagatctggacccaaaagggggaaacatgcctggacccacagactgcgtgagagaaagca
gctggtgatttatgaagagatcagcgaccctgaggaagatgacgagtaa**CTCCCCTGGGGGATACGACACATGCCCTTGATGAGAAGCAGAACGTGGTGACCTTTCACGAACATGGGCATGGCTGCGGCTCCCTCGTCA**

Notes:

-In UPPER CASE is shown SYT sequence

-In lower case is shown SSX1/2 sequence as indicated

-In **BOLD UPPER** case SSX1/2 consensus 3’ UTR as indicated

-In highlighted PURPLE is shown stop codon TAA

-SSX1 Polymorphism highlighted in BLUE

S5C Fig

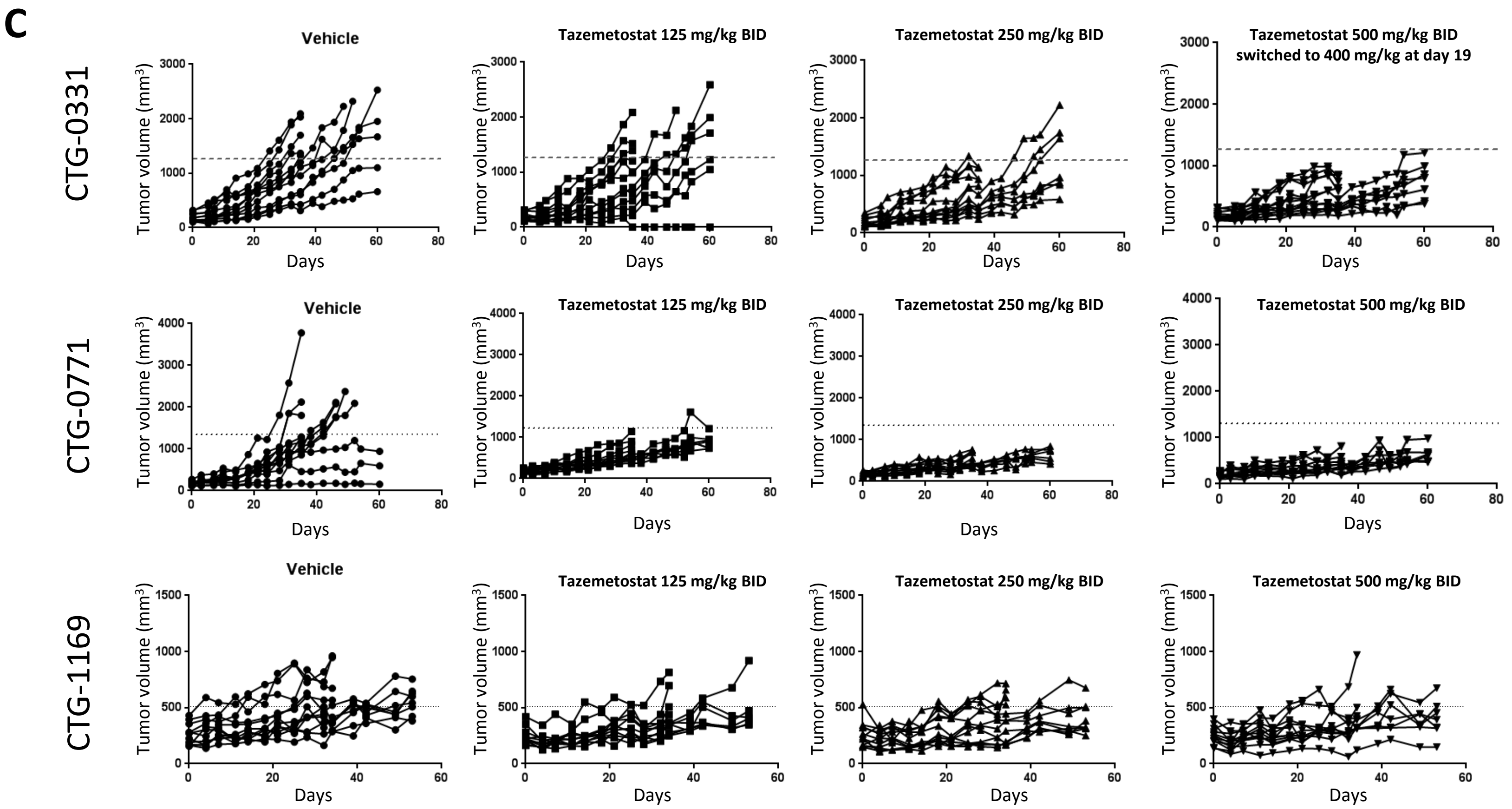
C
CTG-0331
CTG-0771
CTG-1169
Vehicle
Tazemetostat 125 mg/kg BID
Tazemetostat 250 mg/kg BID
Tazemetostat 500 mg/kg BID
switched to 400 mg/kg at day 19
Tazemetostat 500 mg/kg BID
Tumor volume (mm³)
Days

S5D-S5F Figs

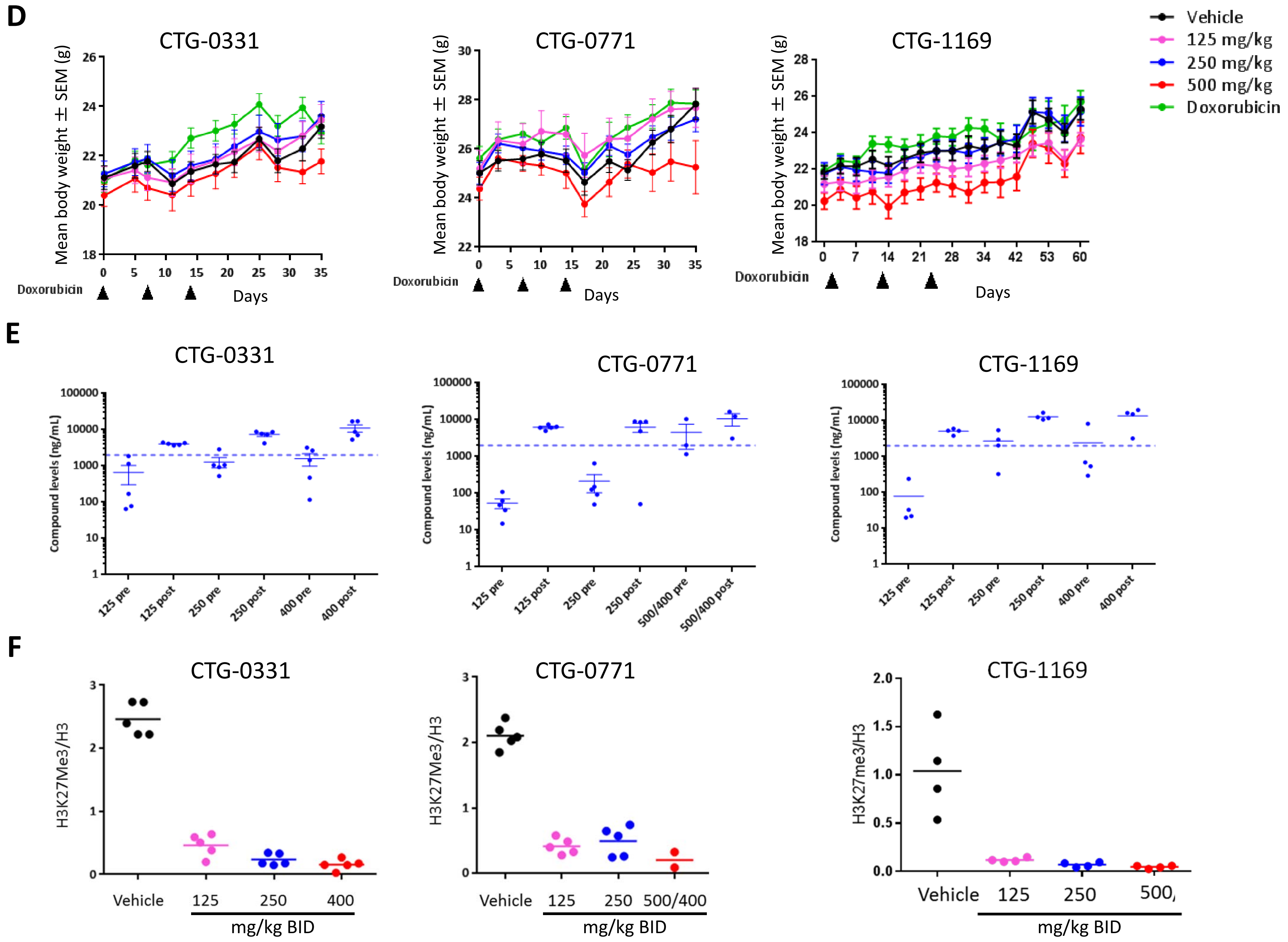
D
CTG-0331
CTG-0771
CTG-1169
Mean body weight ± SEM (g)
Days
Doxorubicin
Vehicle
125 mg/kg
250 mg/kg
500 mg/kg
Doxorubicin
E
CTG-0331
CTG-0771
CTG-1169
Compound levels (ng/mL)
125 pre
125 post
250 pre
250 post
400 pre
400 post
500/400 pre
500/400 post
F
CTG-0331
CTG-0771
CTG-1169
H3K27Me3/H3
H3K27me3/H3
Vehicle
125
250
400
500/400
500,
mg/kg BID

S5G Fig

G

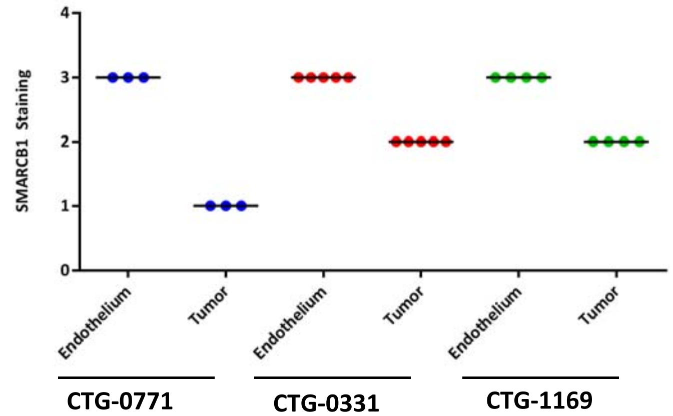
SMARCB1 Staining
4
3
2
1
0
Endothelium
Tumor
Endothelium
Tumor
Endothelium
Tumor
CTG-0771
CTG-0331
CTG-1169

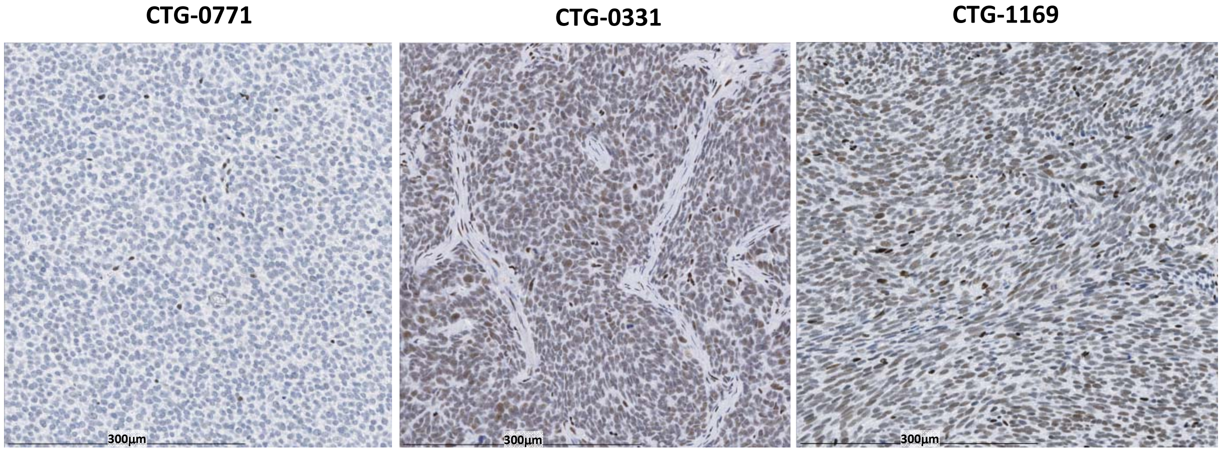
CTG-0771
CTG-0331
CTG-1169
300µm
300µm
300µm

S5H Fig

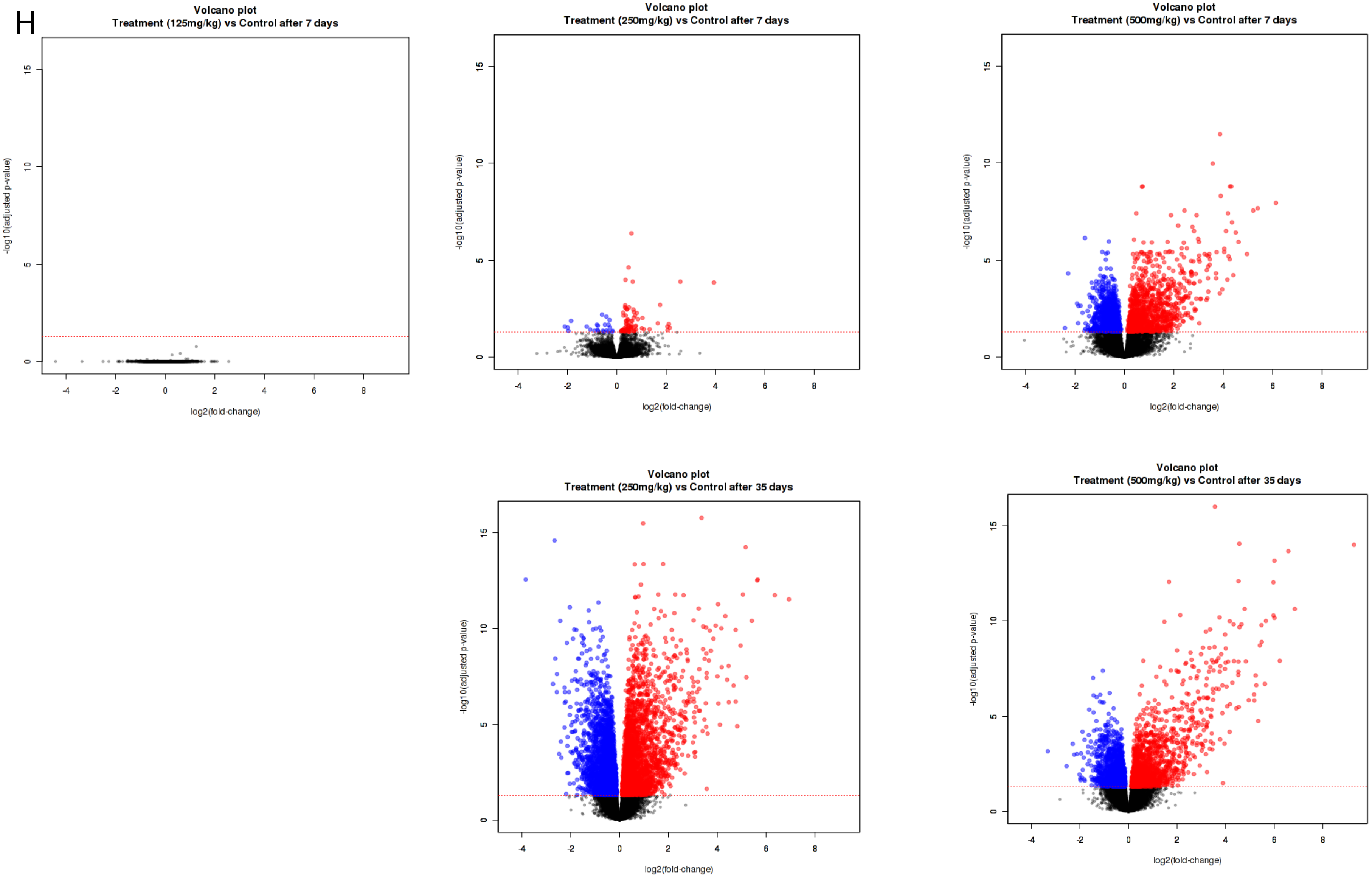
H
Volcano plot
Treatment (125mg/kg) vs Control after 7 days
Volcano plot
Treatment (250mg/kg) vs Control after 7 days
Volcano plot
Treatment (500mg/kg) vs Control after 7 days
Volcano plot
Treatment (250mg/kg) vs Control after 35 days
Volcano plot
Treatment (500mg/kg) vs Control after 35 days
-log10(adjusted p-value)
log2(fold-change)
0
5
10
15
-4
-2
0
2
4
6
8

S5I Fig

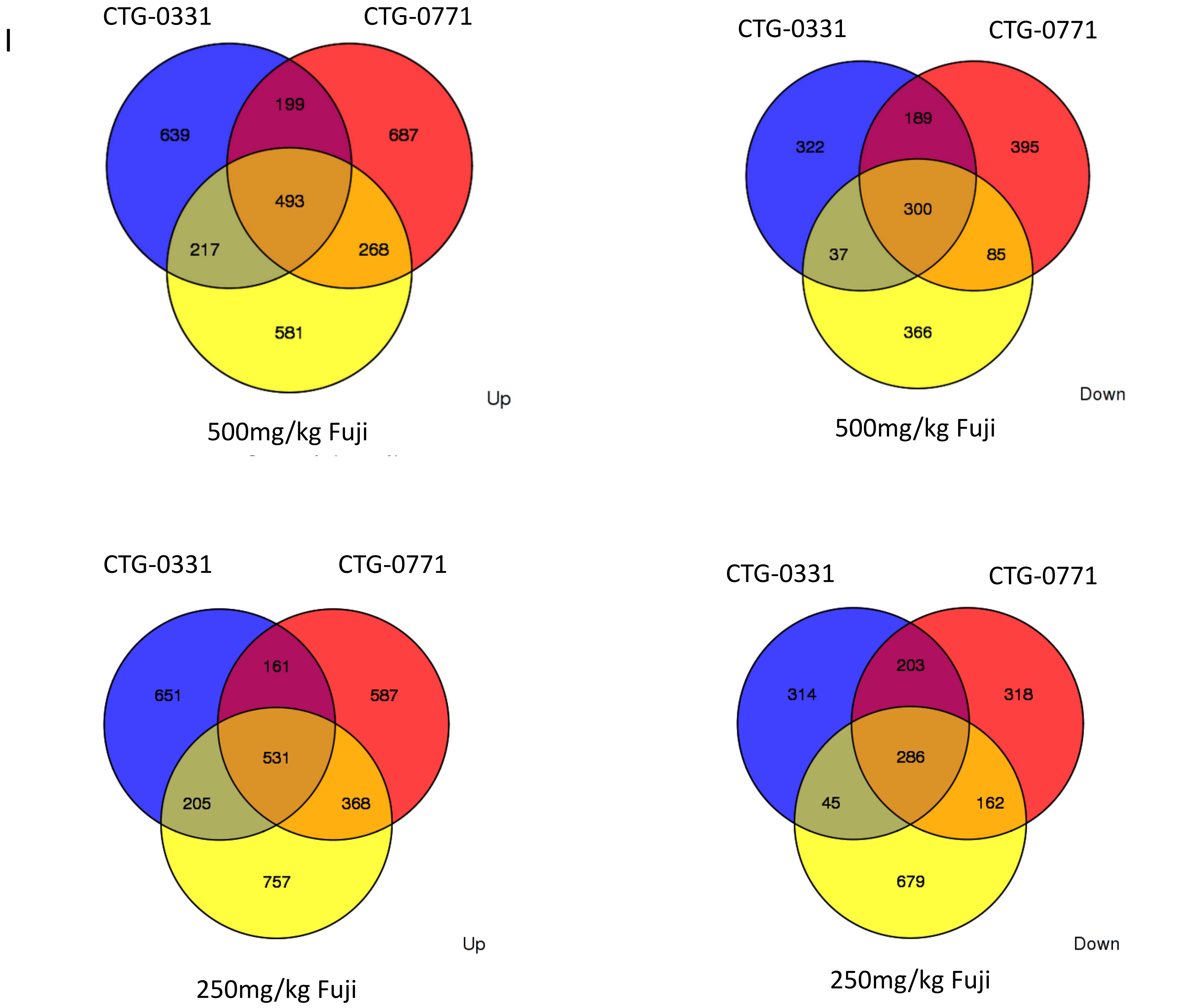
I
CTG-0331
CTG-0771
639
199
687
493
217
268
581
Up
500mg/kg Fuji
CTG-0331
CTG-0771
322
189
395
300
37
85
366
Down
500mg/kg Fuji
CTG-0331
CTG-0771
651
161
587
531
205
368
757
Up
250mg/kg Fuji
CTG-0331
CTG-0771
314
203
318
286
45
162
679
Down
250mg/kg Fuji

S5J Fig

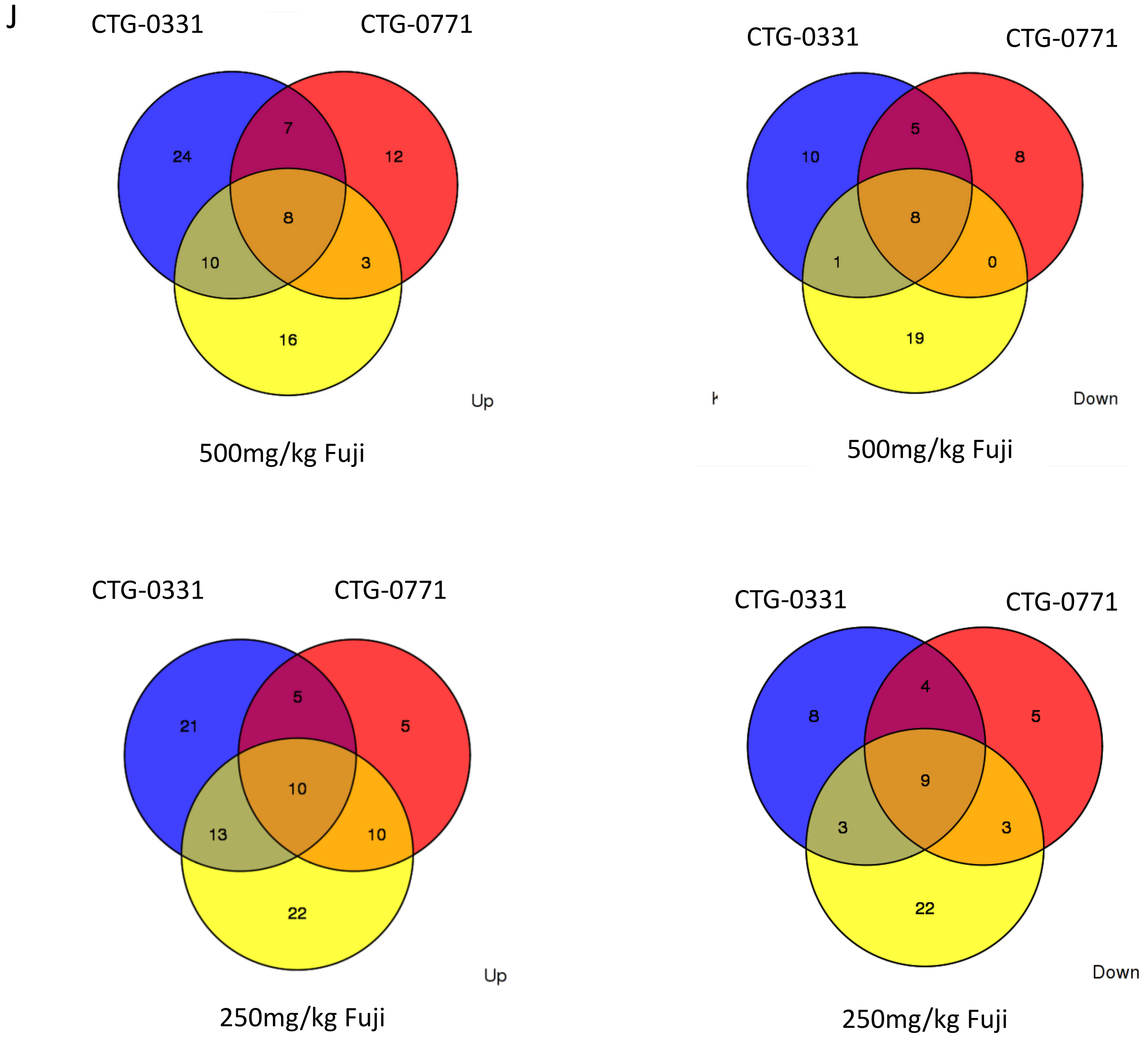
J
CTG-0331
CTG-0771
24
7
12
8
10
3
16
Up
500mg/kg Fuji
CTG-0331
CTG-0771
10
5
8
8
1
0
19
Down
500mg/kg Fuji
CTG-0331
CTG-0771
21
5
5
10
13
10
22
Up
250mg/kg Fuji
CTG-0331
CTG-0771
8
4
5
9
3
3
22
Down
250mg/kg Fuji
