# Supplementary material for: Preclinical Evidence of Anti-Tumor Activity Induced by EZH2 Inhibition in Human Models of Synovial Sarcoma
Source: PLoS One. 2016 Jul 8;11(7):e0158888. doi: 10.1371/journal.pone.0158888 (PMC4938529; doi:10.1371/journal.pone.0158888)
Supplement: S1 Table — (PDF) [file pone.0158888.s006.pdf]

Table S1A: Congruent GO Pathways Upregulated for CTG-0331, CTG-0771 and Fuji 250mg/kg

PathwayID	PathwayName
GO:2001016	positive regulation of skeletal muscle cell differentiation
GO:2000648	positive regulation of stem cell proliferation
GO:2000352	negative regulation of endothelial cell apoptotic process
GO:2000351	regulation of endothelial cell apoptotic process
GO:2000147	positive regulation of cell motility
GO:2000146	negative regulation of cell motility
GO:2000145	regulation of cell motility
GO:2000021	regulation of ion homeostasis
GO:1903034	regulation of response to wounding
GO:1902533	positive regulation of intracellular signal transduction
GO:1902531	regulation of intracellular signal transduction
GO:1901701	cellular response to oxygen-containing compound
GO:1901700	response to oxygen-containing compound
GO:1901699	cellular response to nitrogen compound
GO:1901698	response to nitrogen compound
GO:1900542	regulation of purine nucleotide metabolic process
GO:0098647	collagen beaded filament
GO:0098644	complex of collagen trimers
GO:0098602	single organism cell adhesion
GO:0098552	side of membrane
GO:0098531	transcription factor activity, direct ligand regulated sequence-specific DNA binding
GO:0097485	neuron projection guidance
GO:0097306	cellular response to alcohol
GO:0090287	regulation of cellular response to growth factor stimulus
GO:0090257	regulation of muscle system process
GO:0090132	epithelium migration
GO:0090101	negative regulation of transmembrane receptor protein serine/threonine kinase signaling pathway
GO:0090092	regulation of transmembrane receptor protein serine/threonine kinase signaling pathway
GO:0090090	negative regulation of canonical Wnt signaling pathway
GO:0080134	regulation of response to stress
GO:0072577	endothelial cell apoptotic process
GO:0072511	divalent inorganic cation transport
GO:0072507	divalent inorganic cation homeostasis
GO:0072359	circulatory system development
GO:0072358	cardiovascular system development
GO:0072091	regulation of stem cell proliferation
GO:0072089	stem cell proliferation
GO:0072073	kidney epithelium development
GO:0072001	renal system development
GO:0071944	cell periphery
GO:0071774	response to fibroblast growth factor
GO:0071694	maintenance of protein location in extracellular region
GO:0071692	protein localization to extracellular region
GO:0071495	cellular response to endogenous stimulus
GO:0071417	cellular response to organonitrogen compound
GO:0071407	cellular response to organic cyclic compound
GO:0071396	cellular response to lipid
GO:0071363	cellular response to growth factor stimulus
GO:0071310	cellular response to organic substance
GO:0071300	cellular response to retinoic acid
GO:0071229	cellular response to acid chemical

GO:0070887	cellular response to chemical stimulus
GO:0070848	response to growth factor
GO:0070482	response to oxygen levels
GO:0070169	positive regulation of biomineral tissue development
GO:0070167	regulation of biomineral tissue development
GO:0065009	regulation of molecular function
GO:0061564	axon development
GO:0061448	connective tissue development
GO:0061061	muscle structure development
GO:0061053	somite development
GO:0061041	regulation of wound healing
GO:0061035	regulation of cartilage development
GO:0060828	regulation of canonical Wnt signaling pathway
GO:0060740	prostate gland epithelium morphogenesis
GO:0060575	intestinal epithelial cell differentiation
GO:0060562	epithelial tube morphogenesis
GO:0060560	developmental growth involved in morphogenesis
GO:0060541	respiratory system development
GO:0060538	skeletal muscle organ development
GO:0060537	muscle tissue development
GO:0060512	prostate gland morphogenesis
GO:0060485	mesenchyme development
GO:0060429	epithelium development
GO:0060402	calcium ion transport into cytosol
GO:0060401	cytosolic calcium ion transport
GO:0060349	bone morphogenesis
GO:0060348	bone development
GO:0060341	regulation of cellular localization
GO:0060291	long-term synaptic potentiation
GO:0060284	regulation of cell development
GO:0060173	limb development
GO:0060089	molecular transducer activity
GO:0060070	canonical Wnt signaling pathway
GO:0060047	heart contraction
GO:0060021	palate development
GO:0060004	reflex
GO:0055123	digestive system development
GO:0055117	regulation of cardiac muscle contraction
GO:0055074	calcium ion homeostasis
GO:0055002	striated muscle cell development
GO:0055001	muscle cell development
GO:0051960	regulation of nervous system development
GO:0051899	membrane depolarization
GO:0051716	cellular response to stimulus
GO:0051674	localization of cell
GO:0051336	regulation of hydrolase activity
GO:0051272	positive regulation of cellular component movement
GO:0051271	negative regulation of cellular component movement
GO:0051270	regulation of cellular component movement
GO:0051247	positive regulation of protein metabolic process
GO:0051241	negative regulation of multicellular organismal process
GO:0051239	regulation of multicellular organismal process
GO:0051216	cartilage development

GO:0051174	regulation of phosphorus metabolic process
GO:0051130	positive regulation of cellular component organization
GO:0051093	negative regulation of developmental process
GO:0051049	regulation of transport
GO:0051046	regulation of secretion
GO:0050920	regulation of chemotaxis
GO:0050878	regulation of body fluid levels
GO:0050839	cell adhesion molecule binding
GO:0050801	ion homeostasis
GO:0050795	regulation of behavior
GO:0050794	regulation of cellular process
GO:0050790	regulation of catalytic activity
GO:0050789	regulation of biological process
GO:0050769	positive regulation of neurogenesis
GO:0050767	regulation of neurogenesis
GO:0050730	regulation of peptidyl-tyrosine phosphorylation
GO:0050680	negative regulation of epithelial cell proliferation
GO:0050679	positive regulation of epithelial cell proliferation
GO:0050678	regulation of epithelial cell proliferation
GO:0050673	epithelial cell proliferation
GO:0048878	chemical homeostasis
GO:0048871	multicellular organismal homeostasis
GO:0048870	cell motility
GO:0048858	cell projection morphogenesis
GO:0048856	anatomical structure development
GO:0048839	inner ear development
GO:0048812	neuron projection morphogenesis
GO:0048771	tissue remodeling
GO:0048762	mesenchymal cell differentiation
GO:0048742	regulation of skeletal muscle fiber development
GO:0048738	cardiac muscle tissue development
GO:0048736	appendage development
GO:0048729	tissue morphogenesis
GO:0048706	embryonic skeletal system development
GO:0048705	skeletal system morphogenesis
GO:0048704	embryonic skeletal system morphogenesis
GO:0048701	embryonic cranial skeleton morphogenesis
GO:0048667	cell morphogenesis involved in neuron differentiation
GO:0048646	anatomical structure formation involved in morphogenesis
GO:0048598	embryonic morphogenesis
GO:0048589	developmental growth
GO:0048585	negative regulation of response to stimulus
GO:0048584	positive regulation of response to stimulus
GO:0048583	regulation of response to stimulus
GO:0048568	embryonic organ development
GO:0048562	embryonic organ morphogenesis
GO:0048545	response to steroid hormone
GO:0048534	hematopoietic or lymphoid organ development
GO:0048514	blood vessel morphogenesis
GO:0048468	cell development
GO:0048011	neurotrophin TRK receptor signaling pathway
GO:0048009	insulin-like growth factor receptor signaling pathway
GO:0046903	secretion

GO:0046889 positive regulation of lipid biosynthetic process
GO:0046849 bone remodeling
GO:0046649 lymphocyte activation
GO:0045937 positive regulation of phosphate metabolic process
GO:0045913 positive regulation of carbohydrate metabolic process
GO:0045834 positive regulation of lipid metabolic process
GO:0045785 positive regulation of cell adhesion
GO:0045765 regulation of angiogenesis
GO:0045732 positive regulation of protein catabolic process
GO:0045664 regulation of neuron differentiation
GO:0045661 regulation of myoblast differentiation
GO:0045637 regulation of myeloid cell differentiation
GO:0045598 regulation of fat cell differentiation
GO:0045597 positive regulation of cell differentiation
GO:0045596 negative regulation of cell differentiation
GO:0045444 fat cell differentiation
GO:0045321 leukocyte activation
GO:0045202 synapse
GO:0045121 membrane raft
GO:0044708 single-organism behavior
GO:0044707 single-multicellular organism process
GO:0044700 single organism signaling
GO:0044459 plasma membrane part
GO:0044425 membrane part
GO:0044420 extracellular matrix component
GO:0044304 main axon
GO:0044057 regulation of system process
GO:0043588 skin development
GO:0043583 ear development
GO:0043567 regulation of insulin-like growth factor receptor signaling pathway
GO:0043410 positive regulation of MAPK cascade
GO:0043408 regulation of MAPK cascade
GO:0043406 positive regulation of MAP kinase activity
GO:0043405 regulation of MAP kinase activity
GO:0043401 steroid hormone mediated signaling pathway
GO:0043112 receptor metabolic process
GO:0043062 extracellular structure organization
GO:0043034 costamere
GO:0043009 chordate embryonic development
GO:0042995 cell projection
GO:0042886 amide transport
GO:0042593 glucose homeostasis
GO:0042592 homeostatic process
GO:0042476 odontogenesis
GO:0042474 middle ear morphogenesis
GO:0042472 inner ear morphogenesis
GO:0042471 ear morphogenesis
GO:0042330 taxis
GO:0042327 positive regulation of phosphorylation
GO:0042221 response to chemical
GO:0042110 T cell activation
GO:0042063 gliogenesis
GO:0042060 wound healing

GO:0040017 positive regulation of locomotion
GO:0040013 negative regulation of locomotion
GO:0040012 regulation of locomotion
GO:0040011 locomotion
GO:0040008 regulation of growth
GO:0040007 growth
GO:0038179 neurotrophin signaling pathway
GO:0036477 somatodendritic compartment
GO:0035987 endodermal cell differentiation
GO:0035947 regulation of gluconeogenesis by regulation of transcription from RNA polymerase II promoter
GO:0035637 multicellular organismal signaling
GO:0035556 intracellular signal transduction
GO:0035295 tube development
GO:0035282 segmentation
GO:0035239 tube morphogenesis
GO:0035113 embryonic appendage morphogenesis
GO:0035108 limb morphogenesis
GO:0035107 appendage morphogenesis
GO:0035023 regulation of Rho protein signal transduction
GO:0033993 response to lipid
GO:0033500 carbohydrate homeostasis
GO:0032990 cell part morphogenesis
GO:0032989 cellular component morphogenesis
GO:0032940 secretion by cell
GO:0032879 regulation of localization
GO:0032870 cellular response to hormone stimulus
GO:0032844 regulation of homeostatic process
GO:0032526 response to retinoic acid
GO:0032501 multicellular organismal process
GO:0032331 negative regulation of chondrocyte differentiation
GO:0032330 regulation of chondrocyte differentiation
GO:0032319 regulation of Rho GTPase activity
GO:0032314 regulation of Rac GTPase activity
GO:0032270 positive regulation of cellular protein metabolic process
GO:0032101 regulation of response to external stimulus
GO:0031994 insulin-like growth factor I binding
GO:0031589 cell-substrate adhesion
GO:0031401 positive regulation of protein modification process
GO:0031346 positive regulation of cell projection organization
GO:0031344 regulation of cell projection organization
GO:0031331 positive regulation of cellular catabolic process
GO:0031329 regulation of cellular catabolic process
GO:0031325 positive regulation of cellular metabolic process
GO:0031226 intrinsic component of plasma membrane
GO:0031214 biomineral tissue development
GO:0031175 neuron projection development
GO:0031012 extracellular matrix
GO:0030888 regulation of B cell proliferation
GO:0030879 mammary gland development
GO:0030850 prostate gland development
GO:0030534 adult behavior
GO:0030510 regulation of BMP signaling pathway
GO:0030509 BMP signaling pathway

GO:0030501	positive regulation of bone mineralization
GO:0030500	regulation of bone mineralization
GO:0030425	dendrite
GO:0030336	negative regulation of cell migration
GO:0030335	positive regulation of cell migration
GO:0030334	regulation of cell migration
GO:0030326	embryonic limb morphogenesis
GO:0030324	lung development
GO:0030323	respiratory tube development
GO:0030282	bone mineralization
GO:0030278	regulation of ossification
GO:0030217	T cell differentiation
GO:0030198	extracellular matrix organization
GO:0030178	negative regulation of Wnt signaling pathway
GO:0030111	regulation of Wnt signaling pathway
GO:0030098	lymphocyte differentiation
GO:0030030	cell projection organization
GO:0023057	negative regulation of signaling
GO:0023056	positive regulation of signaling
GO:0023052	signaling
GO:0023051	regulation of signaling
GO:0023014	signal transduction by protein phosphorylation
GO:0022617	extracellular matrix disassembly
GO:0022612	gland morphogenesis
GO:0022610	biological adhesion
GO:0022604	regulation of cell morphogenesis
GO:0022603	regulation of anatomical structure morphogenesis
GO:0021954	central nervous system neuron development
GO:0021542	dentate gyrus development
GO:0021537	telencephalon development
GO:0019932	second-messenger-mediated signaling
GO:0019838	growth factor binding
GO:0019220	regulation of phosphate metabolic process
GO:0019216	regulation of lipid metabolic process
GO:0019199	transmembrane receptor protein kinase activity
GO:0018212	peptidyl-tyrosine modification
GO:0018108	peptidyl-tyrosine phosphorylation
GO:0016477	cell migration
GO:0016337	single organismal cell-cell adhesion
GO:0016055	Wnt signaling pathway
GO:0016049	cell growth
GO:0015833	peptide transport
GO:0014848	urinary tract smooth muscle contraction
GO:0014706	striated muscle tissue development
GO:0014031	mesenchymal cell development
GO:0010975	regulation of neuron projection development
GO:0010959	regulation of metal ion transport
GO:0010906	regulation of glucose metabolic process
GO:0010817	regulation of hormone levels
GO:0010811	positive regulation of cell-substrate adhesion
GO:0010810	regulation of cell-substrate adhesion
GO:0010769	regulation of cell morphogenesis involved in differentiation
GO:0010720	positive regulation of cell development

GO:0010676	positive regulation of cellular carbohydrate metabolic process
GO:0010675	regulation of cellular carbohydrate metabolic process
GO:0010648	negative regulation of cell communication
GO:0010647	positive regulation of cell communication
GO:0010646	regulation of cell communication
GO:0010634	positive regulation of epithelial cell migration
GO:0010632	regulation of epithelial cell migration
GO:0010631	epithelial cell migration
GO:0010629	negative regulation of gene expression
GO:0010604	positive regulation of macromolecule metabolic process
GO:0010595	positive regulation of endothelial cell migration
GO:0010594	regulation of endothelial cell migration
GO:0010562	positive regulation of phosphorus metabolic process
GO:0010464	regulation of mesenchymal cell proliferation
GO:0010463	mesenchymal cell proliferation
GO:0010243	response to organonitrogen compound
GO:0010033	response to organic substance
GO:0010001	glial cell differentiation
GO:0009986	cell surface
GO:0009968	negative regulation of signal transduction
GO:0009967	positive regulation of signal transduction
GO:0009966	regulation of signal transduction
GO:0009952	anterior/posterior pattern specification
GO:0009897	external side of plasma membrane
GO:0009896	positive regulation of catabolic process
GO:0009894	regulation of catabolic process
GO:0009893	positive regulation of metabolic process
GO:0009888	tissue development
GO:0009887	organ morphogenesis
GO:0009792	embryo development ending in birth or egg hatching
GO:0009790	embryo development
GO:0009755	hormone-mediated signaling pathway
GO:0009725	response to hormone
GO:0009719	response to endogenous stimulus
GO:0009653	anatomical structure morphogenesis
GO:0009611	response to wounding
GO:0009605	response to external stimulus
GO:0009214	cyclic nucleotide catabolic process
GO:0008285	negative regulation of cell proliferation
GO:0008201	heparin binding
GO:0008038	neuron recognition
GO:0008016	regulation of heart contraction
GO:0008015	blood circulation
GO:0007626	locomotory behavior
GO:0007610	behavior
GO:0007566	embryo implantation
GO:0007528	neuromuscular junction development
GO:0007519	skeletal muscle tissue development
GO:0007517	muscle organ development
GO:0007507	heart development
GO:0007492	endoderm development
GO:0007423	sensory organ development
GO:0007417	central nervous system development

GO:0007411	axon guidance
GO:0007389	pattern specification process
GO:0007369	gastrulation
GO:0007268	synaptic transmission
GO:0007267	cell-cell signaling
GO:0007266	Rho protein signal transduction
GO:0007178	transmembrane receptor protein serine/threonine kinase signaling pathway
GO:0007169	transmembrane receptor protein tyrosine kinase signaling pathway
GO:0007167	enzyme linked receptor protein signaling pathway
GO:0007166	cell surface receptor signaling pathway
GO:0007165	signal transduction
GO:0007155	cell adhesion
GO:0007154	cell communication
GO:0006942	regulation of striated muscle contraction
GO:0006937	regulation of muscle contraction
GO:0006935	chemotaxis
GO:0006928	movement of cell or subcellular component
GO:0006816	calcium ion transport
GO:0006140	regulation of nucleotide metabolic process
GO:0006112	energy reserve metabolic process
GO:0006109	regulation of carbohydrate metabolic process
GO:0005901	caveola
GO:0005887	integral component of plasma membrane
GO:0005886	plasma membrane
GO:0005615	extracellular space
GO:0005614	interstitial matrix
GO:0005604	basement membrane
GO:0005589	collagen type VI trimer
GO:0005578	proteinaceous extracellular matrix
GO:0005539	glycosaminoglycan binding
GO:0005520	insulin-like growth factor binding
GO:0005518	collagen binding
GO:0005509	calcium ion binding
GO:0005201	extracellular matrix structural constituent
GO:0005178	integrin binding
GO:0005158	insulin receptor binding
GO:0005109	frizzled binding
GO:0005102	receptor binding
GO:0004879	RNA polymerase II transcription factor activity, ligand-activated sequence-specific DNA binding
GO:0004871	signal transducer activity
GO:0004714	transmembrane receptor protein tyrosine kinase activity
GO:0004115	3',5'-cyclic-AMP phosphodiesterase activity
GO:0004114	3',5'-cyclic-nucleotide phosphodiesterase activity
GO:0004112	cyclic-nucleotide phosphodiesterase activity
GO:0003707	steroid hormone receptor activity
GO:0003015	heart process
GO:0003013	circulatory system process
GO:0003008	system process
GO:0003007	heart morphogenesis
GO:0003002	regionalization
GO:0002934	desmosome organization
GO:0002694	regulation of leukocyte activation
GO:0002682	regulation of immune system process

GO:0002520	immune system development
GO:0002286	T cell activation involved in immune response
GO:0002065	columnar/cuboidal epithelial cell differentiation
GO:0002062	chondrocyte differentiation
GO:0002053	positive regulation of mesenchymal cell proliferation
GO:0002028	regulation of sodium ion transport
GO:0002009	morphogenesis of an epithelium
GO:0001968	fibronectin binding
GO:0001965	G-protein alpha-subunit binding
GO:0001944	vasculature development
GO:0001935	endothelial cell proliferation
GO:0001934	positive regulation of protein phosphorylation
GO:0001837	epithelial to mesenchymal transition
GO:0001822	kidney development
GO:0001775	cell activation
GO:0001764	neuron migration
GO:0001756	somitogenesis
GO:0001755	neural crest cell migration
GO:0001706	endoderm formation
GO:0001704	formation of primary germ layer
GO:0001667	ameboidal-type cell migration
GO:0001655	urogenital system development
GO:0001654	eye development
GO:0001649	osteoblast differentiation
GO:0001568	blood vessel development
GO:0001558	regulation of cell growth
GO:0001525	angiogenesis
GO:0001503	ossification
GO:0001502	cartilage condensation
GO:0001501	skeletal system development
GO:0001227	transcriptional repressor activity, RNA polymerase II transcription regulatory region sequence-specific binding
GO:0001101	response to acid chemical
GO:0000982	transcription factor activity, RNA polymerase II core promoter proximal region sequence-specific binding
GO:0000904	cell morphogenesis involved in differentiation
GO:0000902	cell morphogenesis
GO:0000165	MAPK cascade
GO:0000122	negative regulation of transcription from RNA polymerase II promoter
GO:0060548	negative regulation of cell death
GO:0051246	regulation of protein metabolic process
GO:0051128	regulation of cellular component organization
GO:0048732	gland development
GO:0044763	single-organism cellular process
GO:0044699	single-organism process
GO:0043069	negative regulation of programmed cell death
GO:0043066	negative regulation of apoptotic process
GO:0042127	regulation of cell proliferation
GO:0016265	death
GO:0008283	cell proliferation
GO:2000113	negative regulation of cellular macromolecule biosynthetic process
GO:2000026	regulation of multicellular organismal development
GO:1902679	negative regulation of RNA biosynthetic process
GO:0097458	neuron part
GO:0097285	cell-type specific apoptotic process

GO:0072164	mesonephric tubule development
GO:0072163	mesonephric epithelium development
GO:0065008	regulation of biological quality
GO:0065007	biological regulation
GO:0051253	negative regulation of RNA metabolic process
GO:0051172	negative regulation of nitrogen compound metabolic process
GO:0051094	positive regulation of developmental process
GO:0050896	response to stimulus
GO:0050808	synapse organization
GO:0050793	regulation of developmental process
GO:0048869	cellular developmental process
GO:0048731	system development
GO:0048699	generation of neurons
GO:0048666	neuron development
GO:0048565	digestive tract development
GO:0048523	negative regulation of cellular process
GO:0048522	positive regulation of cellular process
GO:0048519	negative regulation of biological process
GO:0048518	positive regulation of biological process
GO:0048513	animal organ development
GO:0045934	negative regulation of nucleobase-containing compound metabolic process
GO:0045892	negative regulation of transcription, DNA-templated
GO:0045595	regulation of cell differentiation
GO:0044767	single-organism developmental process
GO:0043565	sequence-specific DNA binding
GO:0043005	neuron projection
GO:0042325	regulation of phosphorylation
GO:0032502	developmental process
GO:0031327	negative regulation of cellular biosynthetic process
GO:0031324	negative regulation of cellular metabolic process
GO:0030514	negative regulation of BMP signaling pathway
GO:0030182	neuron differentiation
GO:0030155	regulation of cell adhesion
GO:0030154	cell differentiation
GO:0022008	neurogenesis
GO:0019222	regulation of metabolic process
GO:0014070	response to organic cyclic compound
GO:0014033	neural crest cell differentiation
GO:0014032	neural crest cell development
GO:0010605	negative regulation of macromolecule metabolic process
GO:0010558	negative regulation of macromolecule biosynthetic process
GO:0009892	negative regulation of metabolic process
GO:0009890	negative regulation of biosynthetic process
GO:0008284	positive regulation of cell proliferation
GO:0008219	cell death
GO:0007409	axonogenesis
GO:0007399	nervous system development
GO:0007275	multicellular organism development
GO:0007162	negative regulation of cell adhesion
GO:0001936	regulation of endothelial cell proliferation
GO:0001932	regulation of protein phosphorylation
GO:0001823	mesonephros development
GO:0001657	ureteric bud development

GO:0001012	RNA polymerase II regulatory region DNA binding
GO:0000981	RNA polymerase II transcription factor activity, sequence-specific DNA binding
GO:0000977	RNA polymerase II regulatory region sequence-specific DNA binding
